# Sex-specific and shared expression profiles of vulnerability and resilience to trauma in brain and blood

**DOI:** 10.1186/s13293-020-00288-6

**Published:** 2020-03-30

**Authors:** Grace S. Kim, Monica Uddin

**Affiliations:** 1 Neuroscience Program, University of Illinois at Urbana–Champaign, Urbana, IL USA; 2 Medical Scholars Program, University of Illinois College of Medicine at Urbana-Champaign, Urbana, IL USA; 3 Genomics Program, Center for Global Health and Infectious Disease Research, College of Public Health, University of South Florida, 3720 Spectrum Blvd., Ste. 304, Tampa, FL 33612 USA

## Abstract

**Background:**

While post-traumatic stress disorder (PTSD) is defined by behavioral/cognitive symptoms most directly relevant to brain function, it can be considered a systemic disorder characterized by a distinct inability to reinstate homeostasis after trauma.

**Methods:**

In this study, we conducted a secondary analysis of gene expression profiles in key PTSD-relevant tissues, namely blood, amygdala, and hippocampus, from a rat model of PTSD, to identify sex-specific and shared processes associated with individual differences in response to recent trauma exposure.

**Results:**

Our findings suggest both shared and sex-specific mechanisms underlying individual differences associated with vulnerability and resilience to trauma in hippocampus, amygdala, and blood. By disentangling cell composition from transcriptional changes, we found higher proportions of hippocampal oligodendrocytes in the PTSD-like, extreme behavioral response (EBR) group for both sexes and also identified modules for transcriptional activity associated with group differences (i.e., response to trauma) in the hippocampus that appeared to be sex-specific. By contrast, we found prominent sex differences, but no group differences, in amygdalar cell composition, and both shared and sex-specific modules representing PTSD-relevant transcriptional activity in the amygdala. Across amygdala and hippocampus, both sex-specific and shared processes were relevant to an overarching framework for EBR implicating disrupted TNFα/NFκΒ signaling and excitatory/inhibitory imbalance in dysregulated synaptic/structural plasticity with important implications for fear learning and memory. Our main finding in peripheral blood was consistent with the human literature and identified wound healing processes and hemostasis to be upregulated in the resilient, minimal behavioral response (MBR) group across sexes, but disrupted in a sexually dimorphic manner in the EBR group.

**Conclusion:**

In contrast to the varied characterization of the PTSD-like EBR group, characterization of MBR across blood, amygdala, and hippocampus suggests a common theme of upregulated wound healing and extracellular matrix (ECM) remodeling shared between sexes. In all, we identified differential oligodendrocyte proportions in hippocampus between PTSD-like EBR and resilient MBR, and identified processes and pathways that characterize the EBR and MBR-associated transcriptional changes across hippocampus, amygdala, and blood. The sex-specific mechanisms involved in EBR may contribute to the pronounced disparity in risk for PTSD, with women much more likely to develop PTSD.

## Introduction

Identification of robust peripheral markers for post-traumatic stress disorder (PTSD) would be invaluable for developing PTSD management strategies, especially since accessing human brain tissue is often not a viable option. This requires understanding the relationship between brain and peripheral tissue, such as blood, in the context of stress/trauma exposure. Evidence from the past decade has demonstrated a key role for immune dysregulation in PTSD [1, 2]. In fact, while PTSD is defined by behavioral/cognitive symptoms most directly associated with brain function, it can be considered a systemic disorder involving physiological changes throughout the body across all stages of PTSD [3]. PTSD symptoms reflect a distinct inability to reinstate homeostasis after trauma, and they involve bidirectional crosstalk between the brain and the rest of the body, prominently via peripheral blood, which serves as a conduit for neuroendocrine and immune signaling.

Notably, systemic inflammation may underlie the pathophysiology of PTSD, as well as the consistent link between PTSD and chronic medical conditions associated with aging, such as cardiovascular, metabolic, autoimmune, and neurodegenerative diseases [4–8], and other markers of accelerated aging [6, 7, 9–14]. This dysregulated inflammatory state is itself partially coordinated by maladaptive alterations of hypothalamic-pituitary adrenal (HPA) axis activity and sympathetic nervous system (SNS) sensitivity/responsivity [15, 16], which affect both peripheral immune cells in blood and neuroimmune dynamics in brain. Since these systemic regulatory processes coordinate both brain and blood, ultimately, we would like to determine if transcriptomic signatures of peripheral immune status can inform us about neuroinflammation in the brain and corresponding behavior in response to stress. Furthermore, while not extensively studied in the context of stress and psychoneuroimmunology, these regulatory systems, involved in stress and immune response, are known to have prominent sex differences [17–19]. Thus, considering sex-specific mechanisms in cross-tissue investigations is warranted for characterizing the alterations in peripheral and central nervous system immune states (e.g., neuroinflammation), in response to disrupted HPA, SNS, and immune signaling.

Gene expression (GE) profiles serve as a useful biological readout; taking GE “snapshots” across peripheral blood and key brain regions can help identify processes and pathways disrupted in PTSD that are shared across different tissues, particularly between easily accessible peripheral blood and brain. Animal models of PTSD are key resources that provide access to both blood and brain tissues and allow control of stress/trauma exposure. In this study, we draw from a publicly available dataset [20] that models PTSD using a cut-off behavioral criteria, to assign outbred rats as “vulnerable” or “resilient” based on their behavioral response after stress/trauma exposure (i.e., predator scent stress) [21, 22]. This model is able to differentiate individual behavioral response to stress from general stress response by grouping two extremes of behavior using “diagnostic” inclusion-exclusion criteria, to capture PTSD-like (avoidance/hyperarousal) and resilient/resistant [23] phenotypes as groups distinct from the general population (i.e., middle 50%, not included) [24]. By also including unexposed controls, we are able to distinguish: (1) differential response to stress exposure (vulnerable vs. resilient), which is analogous to human studies of PTSD cases and trauma-exposed controls; (2) individual response to stress exposure (vulnerable exposed vs. unexposed or resilient exposed vs. unexposed); and (3) general response to trauma exposure (exposed [vulnerable and resilient] vs. unexposed).

Here, we conduct a secondary analysis of this publically available dataset to examine gene expression profiles of blood, amygdala, and hippocampus from this rat model of PTSD [20, 24]. Our aim is to identify sex-specific and shared pathways associated with group differences in response to recent trauma exposure, in each of these key PTSD-relevant tissues. In addition to capitalizing on the strengths of this animal model, we disentangle transcriptional activity from cell composition (and/or other covariates) in the bulk expression profile and assess the contributions of cell composition and transcriptional activity to group differences in each tissue. In order to gain a systems-level understanding of transcriptional activity occurring in each tissue, we implement an unsupervised co-expression network approach—to identify groups of genes within a network (i.e., gene modules) and explore their association with sex-stratified group. Finally, we examine the identified modules across tissues to look for common themes.

## Methods

### Dataset

Non-normalized microarray datasets, deposited by the Daskalakis et al. (2014) [20] study, were obtained from the NCBI GEO database [25]. They consisted of 47 blood (GSE60280; 8 samples per group per sex, with one sample missing), 30 amygdalar (GSE60302; 5 samples per group per sex), and 30 hippocampal (GSE60303; 5 samples per group per sex) samples derived from a rat model of PTSD (detailed below) and acquired using the Illumina RatRef-12 v1.0 Expression BeadChip Array platform. Details regarding the dataset and sample processing protocols are published in the original paper [20]. All data import, processing, and analyses were conducted in R (version 3.5.1) [26].

### Animal model of PTSD

The animal model of the PTSD used in Daskalakis et al. (2014) [20] was developed by Cohen and Zohar [24] and involved brief exposure (10 min) of adult outbred Sprague-Dawley rats to predator-scent stress (PSS), an ecologically valid stressor that mimics a life-threatening situation for rodents. Seven days after PSS exposure, the animals were tested for behavioral and physiological response to provocation, and those at either end of the response distribution were categorized as being either vulnerable (EBR, extreme behavioral response) or resilient (MBR, minimal behavioral response), based on statistically validated cut-off behavioral criteria (CBC) [21]. The elevated plus-maze (EPM) and acoustic-startle response (ASR) behavioral tests were used to assess anxiety and arousal, respectively. Comparisons of expression profiles were also made against PSS-unexposed controls (CON). Additional details describing the behavioral assessment and sample processing are provided in the Supporting Information file for Daskalakis et al. (2014) [20].

### Quality control and data processing

Minimal probe detection and inter-array correlation (IAC; Pearson’s) were assessed to identify sample outliers. One blood and one hippocampus sample were excluded due to low signal (less than 4000 significantly detected probes at detection *p* value < 0.01). Then, one sample outlier was identified and removed for each tissue dataset, based on low mean IAC calculated for all pairs of samples within each tissue dataset (standard deviations from mean IAC < − 2.5). After sample removal, 45 out of 47 blood samples, 28 out 30 hippocampal samples, and 29 out of 30 amygdalar samples were retained. Table 1 presents the breakdown of samples used in analyses by tissue, group, and sex. Additional information on QC, data processing, and gene annotation steps is provided in Additional file 1: Methods S1.

**Table 1:** Sample characteristics by tissue, sex, and group after processing

Tissue	Group	Count
Hippocampus	EBR	9 (M: 4; F: 5)
	MBR	9 (M: 4; F: 5)
	CON	10 (M: 5; F: 5)
Amygdala	EBR	9 (M: 5; F: 4)
	MBR	9 (M: 5; F: 4)
	CON	10 (M: 5; F: 5)
Blood	EBR	15 (M: 7; F: 8)
	MBR	16 (M: 8; F: 8)
	CON	14 (M: 8; F: 6)

Count contains number of samples included in analyses, after quality control and data processing, by tissue and group. The breakdown by sex is included in parentheses

*M* male, *F* female, *EBR* extreme behavioral response, *MBR* minimal behavioral response, *CON* trauma unexposed controls

### Brain cell proportion and surrogate variable estimation

Since no validated reference datasets were available for peripheral blood and brain cell types in rats, cell estimation was attempted using marker sets constructed in other species. Brain cell subtypes were estimated using the *BRETIGEA* package, which includes well-conserved consensus brain cell marker sets identified across mouse and human datasets [27] and implements a singular value decomposition approach (SVD), based on CellCODE [28]. This marker set from combined human and mouse measurements contains 1000 markers each for six brain cell types: astrocytes (ast), endothelial cells (end), microglia (mic), neurons (neu), oligodendrocytes (oli), and oligodendrocyte progenitor cells (opc). Of these, only markers expressed in each brain dataset were used for cell estimation (Table 2).

**Table 2:** Brain cell subtype markers used for cell proportion estimates

Tissue	ast	end	mic	neu	oli	opc
Hippocampus	386	352	338	476	460	334
Amygdala	392	372	353	497	468	349

Table reports the number of markers/genes expressed out of 1000 markers per cell type, curated in *BRETIGEA*. These markers were used for cell proportion estimates.

*ast* astrocytes, *end* endothelial cells, *mic* microglia, *neu* neurons, *oli* oligodendrocytes, *opc* oligodendrocyte progenitor cells

Leukocyte cell estimation was attempted using a recently published mouse reference signature matrix, consisting of 511 distinguishing genes for 25 immune cell types [29], modeled after the human LM22 signature matrix constructed for use with the CIBERSORT [30] deconvolution approach. However, this reference dataset was inadequate for deriving reliable leukocyte cell estimates: only 142 out of the 511 signature genes were expressed in our blood dataset. Comparison between mouse and rat genomes has previously revealed significant genomic differences in immune system-related genes between the two rodent models [31]. In addition, known differences in inflammatory system function exist even between mouse strains, further complicating comparisons between the two species [32]. Compared to mice, rats have higher evolutionary rates for immune-related genes and thus possess some genes not found in mouse [31]. Thus, cell estimates were only calculated for brain tissues (i.e., hippocampus and amygdala). Standard statistical approaches were implemented to test for differences in cell composition in brain tissues (see Additional file 1: Methods S1 for more details).

In addition to estimation of brain cell proportions, we conducted surrogate variable analysis [33] on processed expression datasets to derive surrogate variables (SVs) for each tissue, while accounting for sex-stratified group, defined as each individual response category by sex (six levels, e.g., female EBR, male MBR, female CON). There were four SVs identified in hippocampus, five SVs identified in amygdala, and nine SVs identified in blood. Computed SVs were used to account for expression heterogeneity associated with technical and biological artifacts, and are assumed to proxy contributions of cell composition to expression heterogeneity (among other latent sources of confounding) [34]. SV-based analyses were used to corroborate findings based on cell-adjusted analyses in brain-related tissues, and represent our primary analyses in blood.

### Differential expression and gene set enrichment analyses in blood

Since we were not able to estimate cell proportions in blood and had more samples in the blood dataset than in datasets from brain tissues, we conducted differential expression analyses in blood, to cross-check with network analyses (see Additional file 1: Methods for more details).

### Gene co-expression network analysis

Signed co-expression gene network construction and gene module discovery were conducted using the *CEMiTool* package [35], which implements a novel soft threshold (β) selection algorithm, distinct from the original weighted gene co-expression network analysis (WGCNA) approach [36] (refer to Additional file 1: Methods for more details). Unsupervised network analyses in brain tissues were conducted on both SV and cell proportion-adjusted expression data, while analyses in blood were conducted on SV-adjusted data only. Network analyses were also conducted on unadjusted expression data for all tissues for comparison, to infer whether modules represented general processes independent of cell proportions or if they may be linked to cell proportions and other covariates. All analyses were conducted on the full dataset first, followed by sex-stratified datasets, in order to identify modules that are sex-specific, or shared between sexes, consistently across multiple analytic frameworks.

Gene set analyses were conducted for MSigDB v6.2 gene set collections (not including cancer-specific collections) [37, 38] using hypergeometric testing (over-representation analysis) as implemented in *clusterProfiler* [39], for modules of interest identified in gene co-expression network analyses (*adj p* < 0.05; BH *p* value adjustment; gene set with 10–500 genes; qval cutoff 0.2). To identify potential hubs, module graphs were constructed for protein-protein interactions (PPI) from the human PPI dataset on BioGRID [40, 41]. Additional file 2 (available from the corresponding author upon request) contains all gene co-expression network analysis and gene set analysis results, including CEMiTool reports for all analyses with modules detected and tabulated results for gene set analyses of all MSigDB collections with significant results.

## Results

### Higher proportions of hippocampal oligodendrocytes observed in EBR compared to MBR and CON groups, in both sexes

**Fig. 1:**
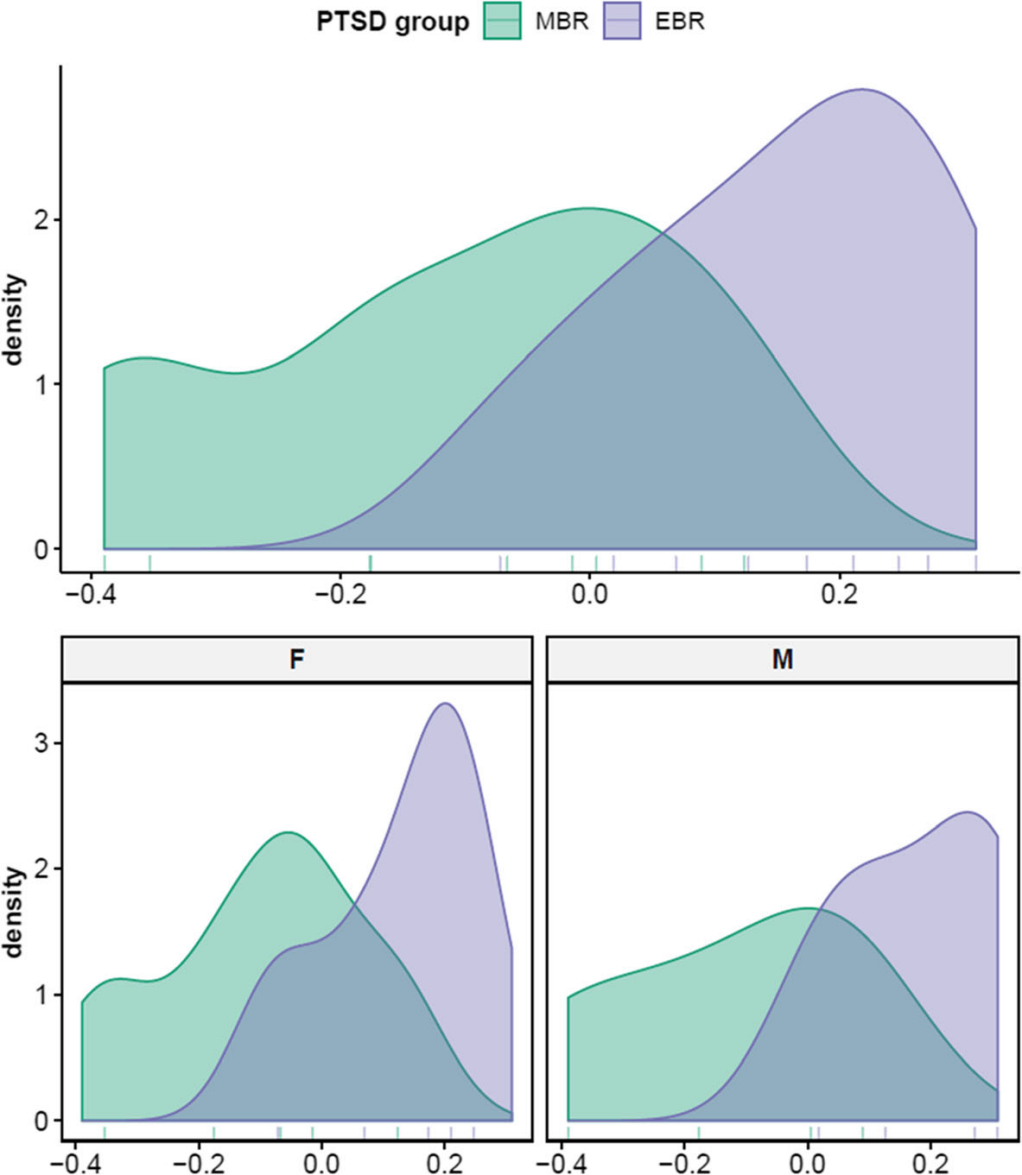
Density plots of hippocampal oligodendrocyte estimates in EBR (purple) and MBR (green) groups show significantly higher proportions in EBR compared to MBR group in both sexes (top). Sex-stratified density plots (bottom) confirm this group difference is not sex-specific. EBR extreme behavioral response (PTSD-like), MBR minimal behavioral response (resilient)

Non-parametric Mann-Whitney *U* test revealed higher median proportions of hippocampal oligodendrocytes in the extreme behavioral response (EBR; PTSD-like) group compared to the minimal behavioral response (MBR; resilient) group in both sexes, *Z* = − 2.78, *Holm-adj p* = 0.024, *r* = 0.66 (Fig. 1). ANCOVA testing the effects of group and sex confirmed significant group differences (EBR vs MBR), *F*(1,15) = 11.4, *p* = 0.004, and no significant sex differences, *F*(1,15) = 0.05, *p* = 0.82, in oligodendrocyte proportions (Table 3). A small, yet significant, difference in hippocampal oligodendrocyte proportions was also observed between EBR and trauma-unexposed control (CON) groups by Kruskal-Wallis, *H*(2) = 9.3, *p* = 0.01, and post hoc Dunn test (Table 4). No significant difference was observed between MBR and CON groups.

**Table 3:** ANCOVA table—hippocampal oligodendrocytes EBR vs. MBR (*n* = 18)

Terms	Sum of squares	*df*	Mean square	*F*	*p*	Partial η^2^
PTSD group	0.297	1	0.297	11.410	0.004***	0.432
Sex	0.001	1	0.001	0.053	0.822	0.004
Residuals	0.390	15	0.026			

***p < 0.005

**Table 4:** Dunn test for Kruskal-Wallis multiple comparison—hippocampal oligodendrocytes

Comparison	*Z*	*p* (*unadj*)	*p* (*adj*)	*r*^2^
CON–EBR	− 2.31	0.021	0.042	0.28
CON–MBR	0.66	0.508	0.508	0.02
EBR–MBR	2.89	0.004	0.011	0.46

*p* values adjusted with the Holm method across group comparisons

Direction of effect for EBR vs CON comparison is the inverse of CON vs EBR comparison

Estimate of percentage variance explained *r*^2^, is calculated from the *Z*-score

**Fig. 2:**
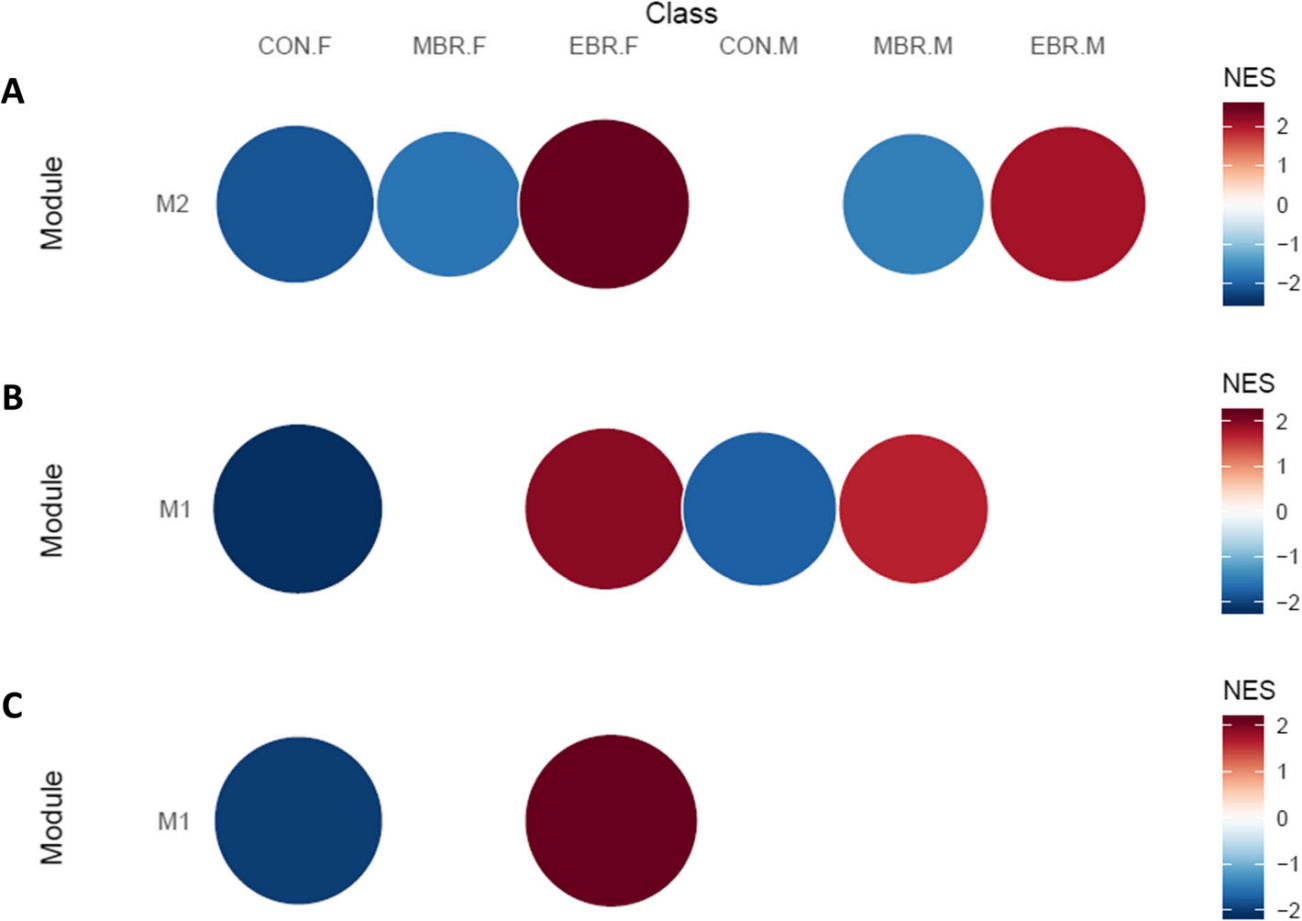
Module enrichment plot for PTSD-relevant modules identified in network analyses of hippocampus. Analyses on full expression dataset (both sexes) that has been **a** unadjusted for cell proportion estimates or surrogate variable (SV) estimates; **b** adjusted for cell proportion estimates; **c** adjusted for SV estimates. **a** Unadjusted: Module M2 consists of 104 genes; upregulation in EBR and downregulation in MBR groups in both sexes, and downregulation in female CON group. **b** Cell-adjusted: Module M1 consists of 165 genes; upregulation in female EBR and male MBR groups with relative downregulation in CON group in both sexes. **c** SV-adjusted: Module M1 consists of 81 genes; upregulation in female EBR and downregulation in female CON group. The size and intensity of the circles correspond to the normalized enrichment score (NES) for the module in each sex-stratified group (normalized by the number of genes in the module)

Additionally, unsupervised network analysis of hippocampal expression levels before cell subtype adjustment identified a gene module significantly associated with upregulation in EBR and downregulation in MBR groups of both sexes, as well as downregulation in the female CON group (M2: 104 genes; Fig. 2a). While the module was not significantly enriched for GO terms, in the curated chemical and genetic perturbation gene set collection (C2 CGP) from the MSigDB database, the top hit enriched in this EBR–MBR module was for oligodendrocyte markers (*adj p* = 0.009; 7 out of 45 genes in *LEIN_OLIGODENDROCYTE_MARKERS*). Comparison of modules from network analyses before and after cell subtype adjustment confirmed the module enriched for oligodendrocyte markers was lost after adjustment, suggesting the enrichment was related to hippocampal cell proportions. Together, results from cell composition and unsupervised network analyses suggest that an increase in hippocampal oligodendrocyte proportions occur in the PTSD-like EBR group, but not the resilient MBR group, in response to trauma exposure. Group differences were not detected in any other hippocampal cell subtype.

### PTSD-relevant hippocampal gene expression modules are sex-specific

Network analyses conducted on the full, cell-adjusted hippocampal expression data identified a module associated with upregulation in the female EBR group, weaker upregulation in the male MBR group, and downregulation in the unexposed CON group from both sexes (M1: 165 genes; Fig. 2b). Top GO terms enriched in this module were cellular response to endogenous stimulus, response to organic cyclic compound, and response to lipid. Relatedly, top hits from other gene set collections identified enrichment for genes upregulated after stimulation with NRG1 and EGF in the C2 CGP collection and TNFα signaling via NFκB pathway in the MSigDB Hallmark collection, implicating activation of ligand-receptor signaling as one possible mechanism for significant processes identified among GO terms. While there were no other modules based on adjusted hippocampal data to confirm the MBR–CON comparison in males, the EBR–CON comparison in females was confirmed in modules identified from SV-adjusted hippocampal data (female subset and full set). The EBR–CON module identified from the female subset of SV-adjusted hippocampal data (M2: 144 genes; Additional file 1: Fig. S1) was significantly enriched for all the top gene sets identified with the M1 module from cell-adjusted hippocampal data (Additional file 1: Table S1) and the female EBR–CON module based on the full SV-adjusted dataset (M1: 81 genes; Fig. 2c) was significantly enriched for the top hallmark and C2 CGP terms, but not GO terms. Of note, the gene set for genes upregulated after NRG1 stimulation was identified as the top hit in the C2 CGP collection in all three EBR–CON modules and module gene members (i.e., genes in the module that overlap with genes in gene set) in the EGF signaling gene set were a subset of module gene members in the NRG1 signaling gene set. In summary, analyses of both cell and SV-adjusted hippocampal data provided consistent evidence implicating upregulation of genes after NRG1 stimulation, and TNFα signaling via NFKΒ pathway in the female EBR group, but not in the male EBR group. Results from sex-stratified analyses on SV-adjusted data in hippocampal tissue are presented in Additional file 1: Results.

### Prominent sex differences in amygdalar cell composition is accompanied by both shared and sex-specific PTSD-relevant expression modules

**Fig. 3:**
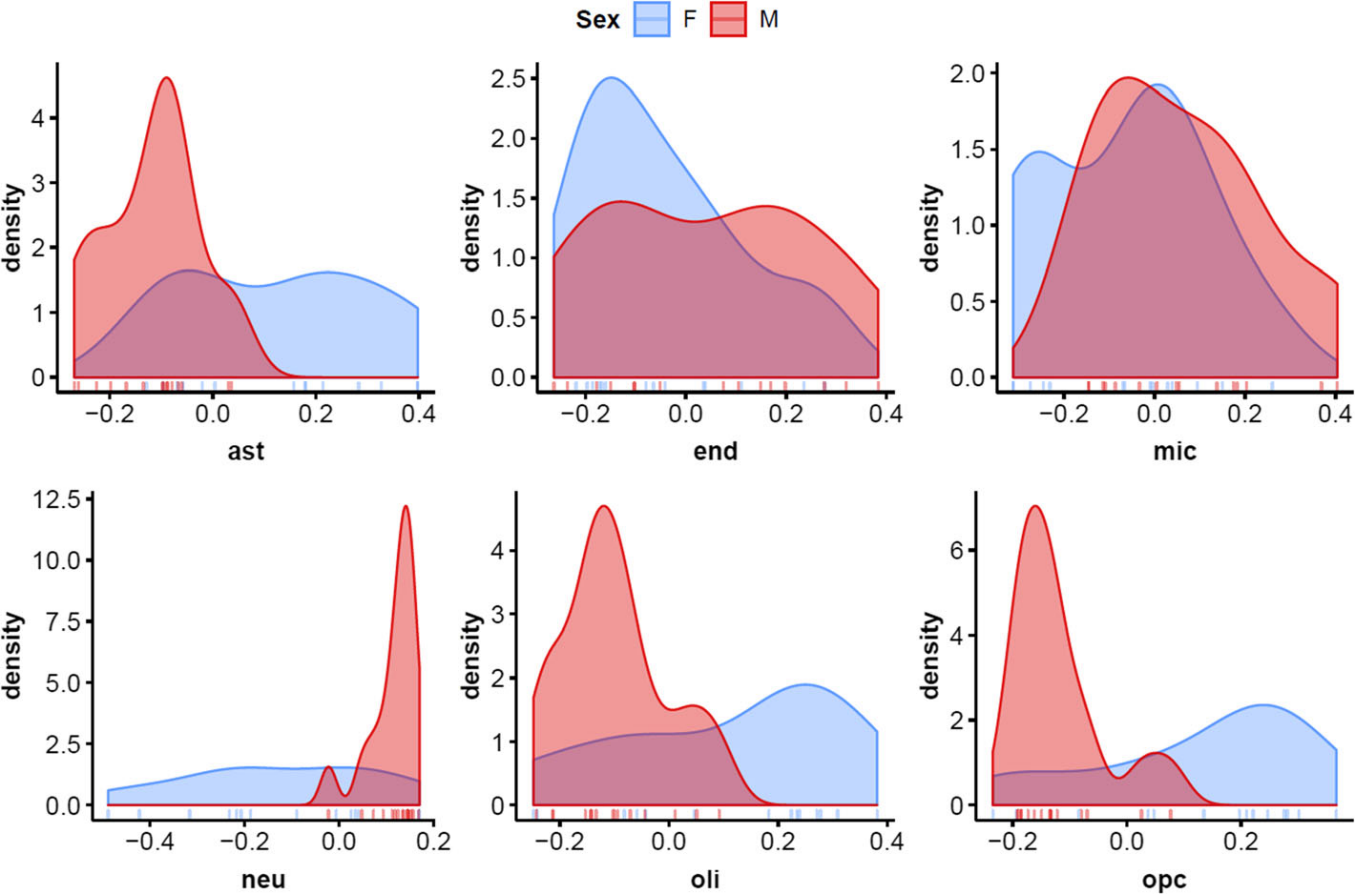
Density plots of amygdalar cell subtypes by sex, show prominent sex differences amygdalar cell composition, significant in astrocytes (ast), neurons (neu), oligodendrocytes (oli), and oligodendrocyte progenitor cells (opc), with females (blue) showing broad distributions (greater variance) and males (red) displaying sharp peaks. Males had relatively higher neu proportion and lower ast, oli, opc proportions

While no significant group differences were detected for any cell subtype in the amygdala (*Holm-adj p* < 0.05), prominent sex differences were observed in the distribution of cell subtype proportions, based on Levene’s and Kolmogorov-Smirnov tests (Table 5). Females, regardless of group, showed significantly greater variance in distribution of relative cell proportions for astrocytes, neurons, oligodendrocytes, and oligodendrocyte progenitor cells, compared to males (Fig. 3).

**Table 5:** Sex differences in distribution of amygdalar cell subtype proportions

Cell subtype	Levene’s test for equality of variances		Two-sample Kolmogorov-Smirnov test	
	*F*	*p*	*D*	*adj p*
ast	7.92	0.009	0.652	0.01
end	1.92	0.18	0.319	0.52
mic	0.011	0.92	0.357	0.52
neu	23.28	4.87e–05	0.790	4.82e–04
oli	5.47	0.027	0.571	0.037
opc	5.18	0.005	0.719	0.003

In two-sample Kolmogorov-Smirnov test, *p* values were adjusted using Holm’s method across cell subtypes. Nominal *p* value is shown for Levene’s test (df 1,27)

*ast* astrocytes, *end* endothelial cells, *mic* microglia, *neu* neurons, *oli* oligodendrocytes, *opc* oligodendrocyte progenitor cells

**Fig. 4:**
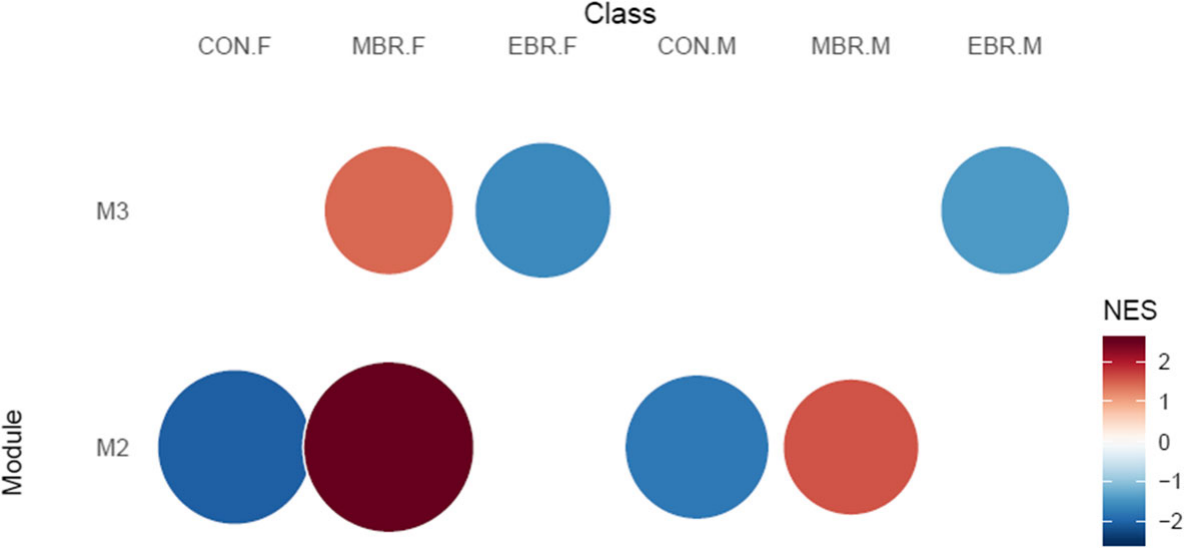
Module enrichment plot for PTSD-relevant modules identified in network analysis of cell-adjusted amygdalar expression levels (both sexes). Module M2 (bottom row) consists of 59 genes and is associated with upregulation in MBR and relative downregulation in CON, in both sexes. Module M3 (top row) consists of 55 genes and is weakly associated with downregulation in EBR groups of both sexes and upregulation in the female MBR group. The size and intensity of the circles correspond to the normalized enrichment score (NES) for the module in each class (normalized by the number of genes in the module)

**Fig. 5:**
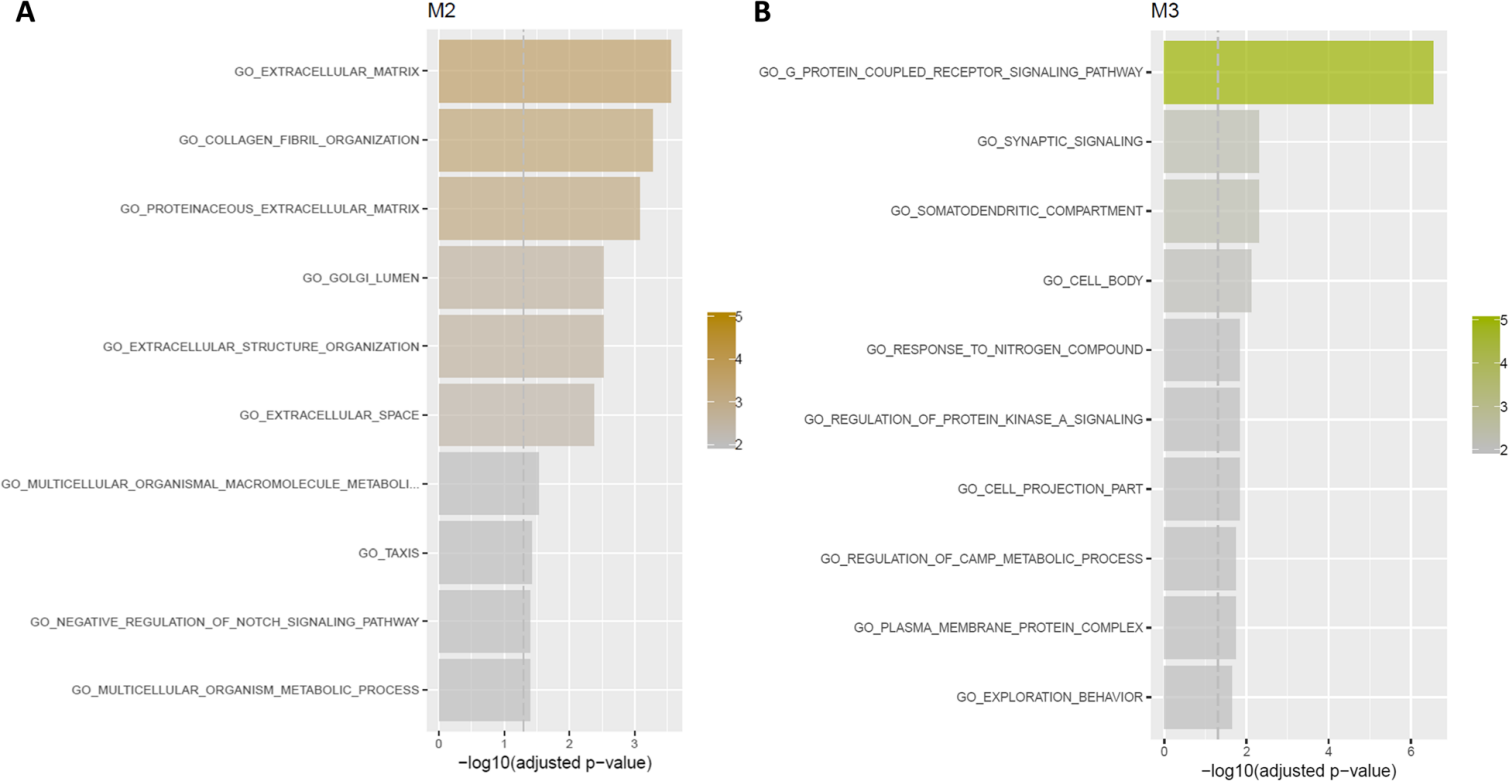
Barplot for top GO terms enriched in **a** M2 and **b** M3 modules identified in network analysis of cell-adjusted amygdalar expression levels. *x*-axis and color transparency display − log_10_ of the Benjamini-Hochberg (BH)-adjusted *p* value. Dashed vertical line indicates BH-adjusted *p* value threshold of 0.05

**Fig. 6:**
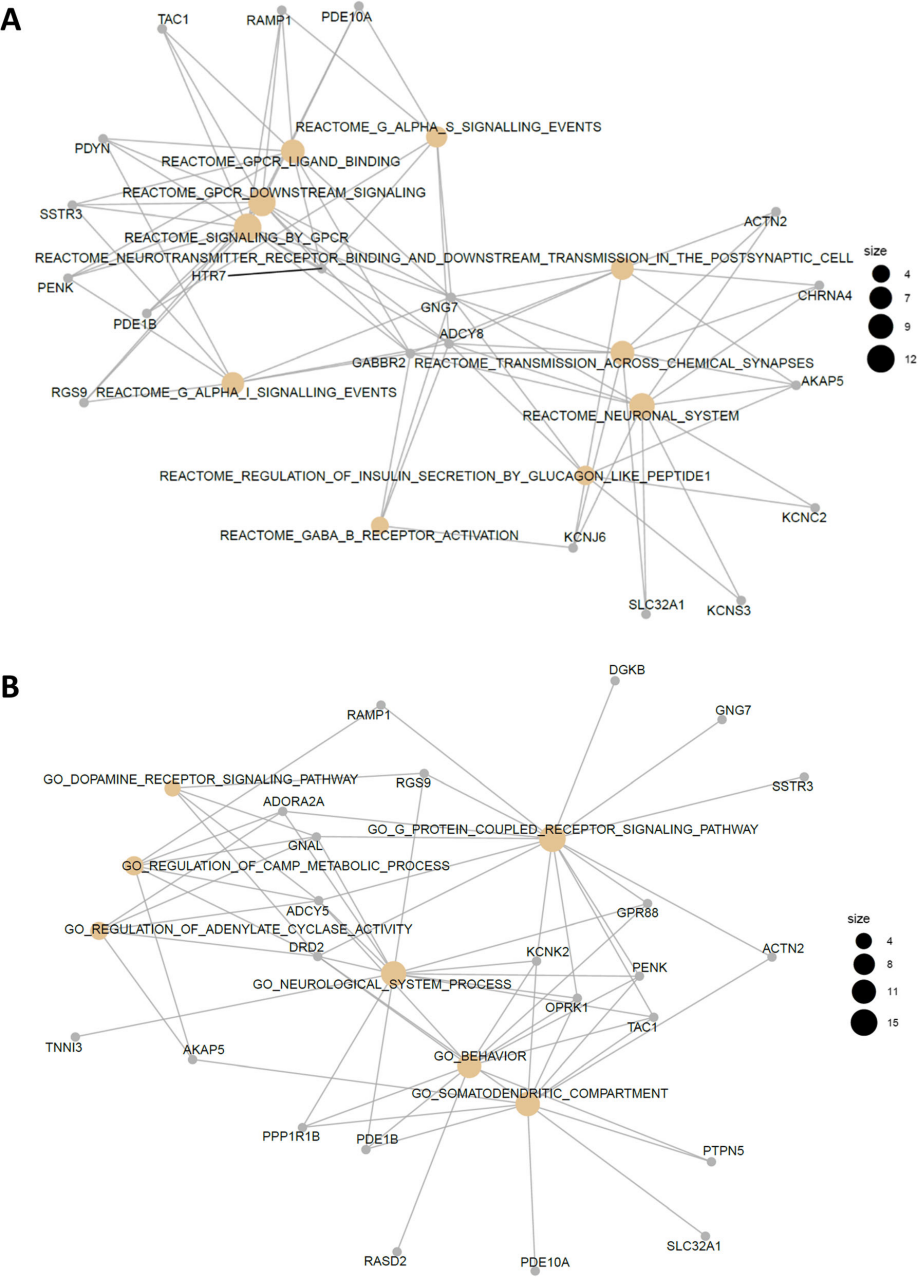
Gene-concept network plot for **a** top REACTOME terms enriched in M3 module of cell-adjusted amygdalar expression levels (55 genes), associated with downregulation in the EBR group for both sexes, with relative upregulation in the female MBR group; **b** top GO terms enriched in M2 module derived from female subset of cell-adjusted amygdala (40 genes). M2 is upregulated in MBR and downregulated in EBR, in females. Size of circles representing terms refers to gene count.

Network analysis of amygdalar expression levels after cell subtype adjustment identified three modules putatively associated with group, either shared between sexes or sex-specific (Fig. 4). Module M2 (59 genes) was associated with upregulation of MBR compared to CON in both sexes, and was enriched for GO terms related to extracellular matrix (ECM), including collagen fibril organization (Fig. 5a), which were corroborated by significant enrichment to matrisome-related terms in the C2 CP collection and ECM organization among REACTOME pathways. Module M3 (55 genes) was weakly associated with downregulation in EBR groups of both sexes and upregulation in the female MBR group. The top GO terms enriched in this module were for GPCR signaling pathway, synaptic signaling and somatodendritic compartment (Fig. 5b). Enrichment results in the REACTOME pathway collection corroborated significant GO hits and suggested enrichment for regulation of insulin secretion by glucagon-like peptide1 and GABAβ receptor activation (Fig. 6a). In all, cell-adjusted network analyses suggest significant upregulation of processes involved in ECM organization in the MBR relative to unexposed CON, and downregulation of GPCR and synaptic signaling in the EBR group in the amygdala, which is shared by both sexes. However, we note downregulation of these pathways/processes in EBR is differentiated from the MBR group only in females.

Sex-stratified network analyses on cell-adjusted amygdalar expression levels did not identify any notable PTSD-relevant module in the male subset but identified one module in the female subset associated with upregulation in the MBR group and downregulation in the EBR group (M2: 40 genes). Top GO terms enriched in this module were GPCR signaling pathway, neurological system process, and behavior (Fig. 6b); these top GO hits were corroborated by significantly enriched terms among REACTOME gene sets. Additionally, the module was enriched for *BRAIN_HCP_WITH_H3K4ME3_AND_H3K27ME3* (C2 CGP; *adj p* = 0.0003; 13/422 genes). Results from analyses on SV-adjusted data in amygdalar tissue are presented in Additional file 1: Results.

### Wound-healing processes in blood are upregulated in MBR across sexes, but sexually dimorphic in EBR

**Fig. 7:**
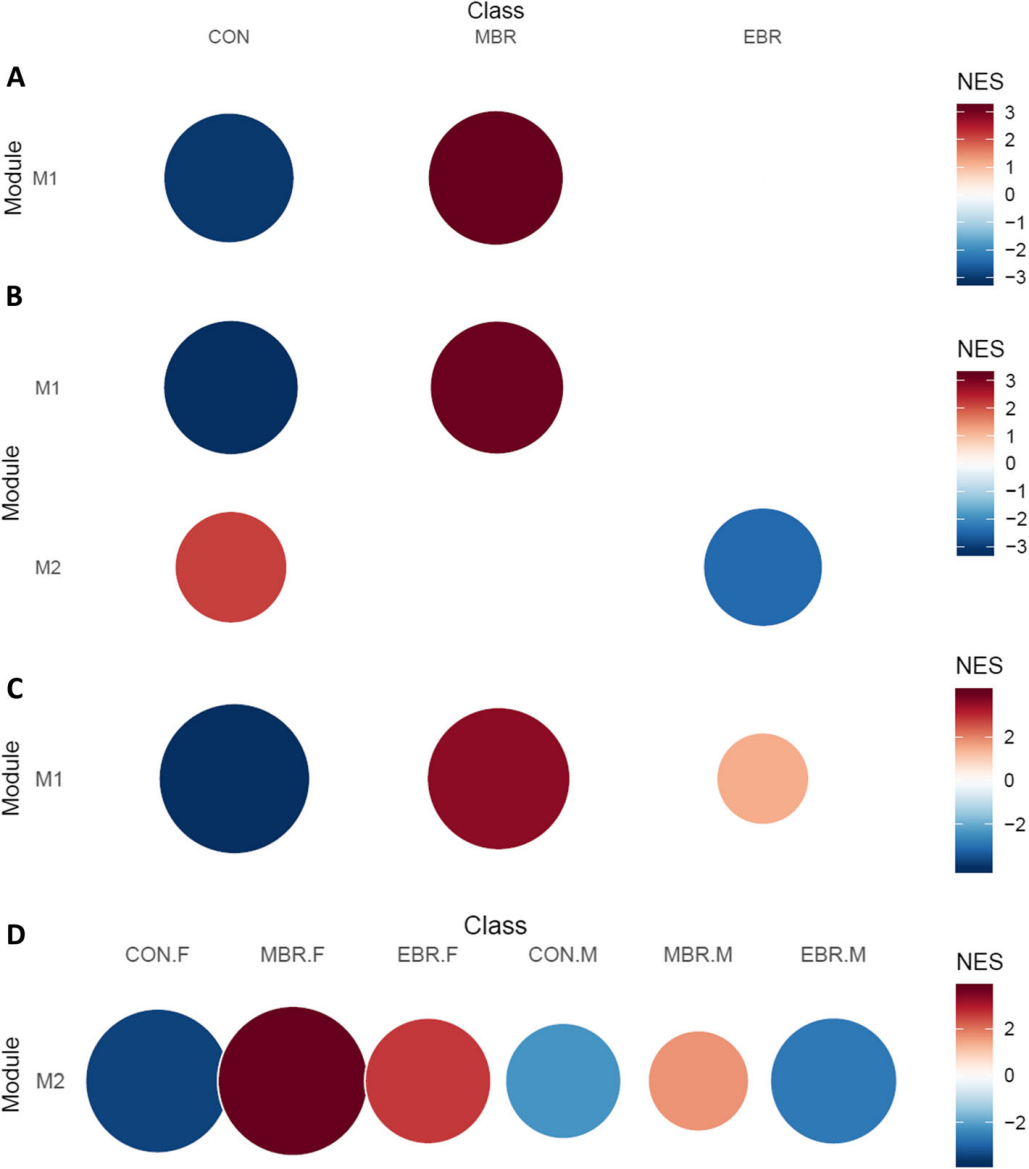
Module enrichment plot for modules identified in blood-based network. Sex-stratified analyses of SV-adjusted data in **a** males and **b** females identified a shared module (M1) upregulated in MBR relative to CON. This module was also identified in un-adjusted data in the **c** female subset and **d** full set (both sexes). Notably, this module also revealed sex-specific group differences in the EBR group and a second module was identified in the SV-adjusted female subset relevant to downregulation in female EBR, relative to CON. The size and intensity of the circles correspond to the normalized enrichment score (NES) for the module in each class (normalized by the number of genes in the module)

**Fig. 8:**
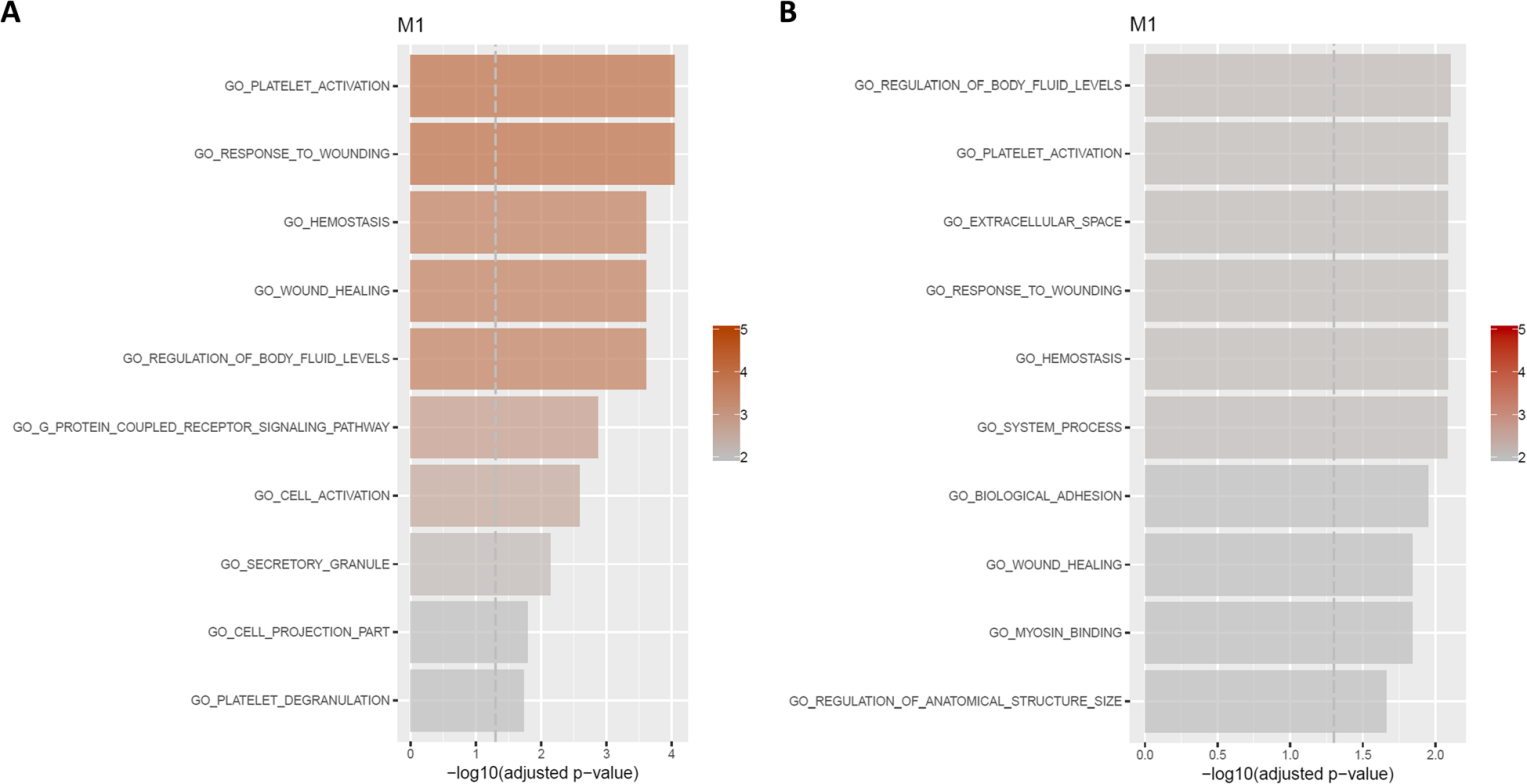
Barplot for top GO terms enriched in M1 modules identified in sex-stratified network analyses of SV-adjusted blood expression levels in **a** males and **b** females. Both modules were upregulated in the MBR group compared to the unexposed CON group. *x*-axis and color transparency display − log_10_ of the Benjamini-Hochberg (BH)-adjusted *p* value. Dashed vertical line indicates BH-adjusted *p* value threshold of 0.05

**Fig. 9:**
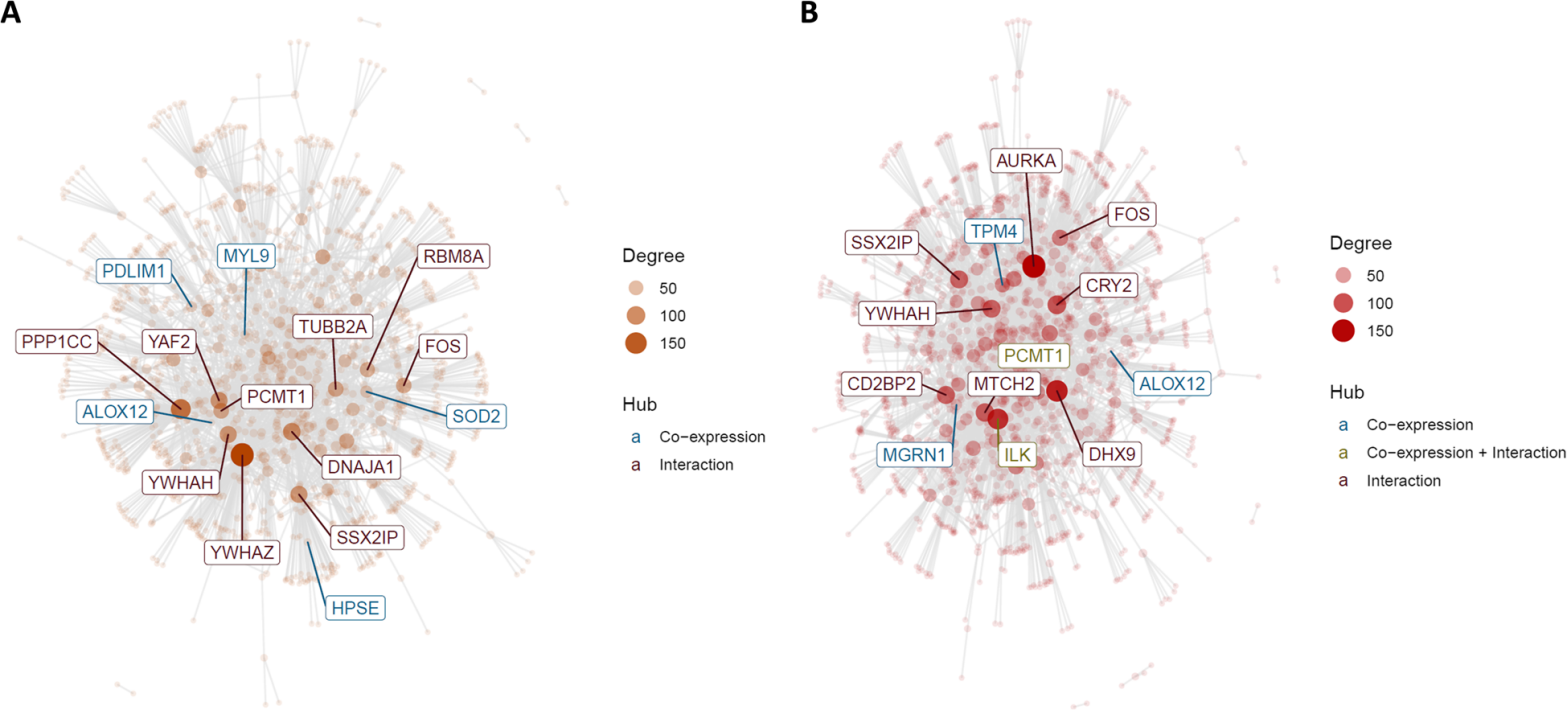
Interaction networks for M1 modules identified in sex-stratified network analyses of SV-adjusted blood expression levels in **a** males and **b** females. Both modules were upregulated in the MBR group compared to the unexposed CON group. Circles identify hub genes in the module and color of their labels indicate nature of associations (i.e., whether association is based on co-expression or interaction between gene products, based on human PPI dataset on BioGRID [40, 41])

While no modules were identified on the full SV-adjusted data, sex-stratified network analyses of SV-adjusted blood expression levels (Fig. 7a, b) identified a module upregulated in the MBR group compared to the unexposed CON group in both males (M1: 134 genes) and females (M1: 186 genes). In both sexes, the module was significantly enriched for GO terms relevant to wound healing, such as platelet activation, response to wound healing, and hemostasis, as well as regulation of body fluid levels (Fig. 8), and these terms were corroborated in other gene set collections for platelet-specific genes (C2 CGP, top hit in both), hemostasis (REACTOME). Interaction networks constructed from sex-stratified SV-adjusted blood expression levels identified *FOS*, *ALOX12*, *PCMT1*, *YWHAH*, and *SSX2IP* as common hub genes in both male and female subsets (Fig. 9).

Sex-specific enrichment was also observed in this MBR–CON module. In males, the module was significantly enriched for GPCR signaling pathway and this was corroborated by significant hits from the REACTOME gene set collection. Additionally, the top hit among KEGG pathways was vascular smooth muscle contraction, which had 7 out of 39 genes in common with the male MBR–CON module. Of these, *PPP1CC* was identified as a significant interaction hub gene in the module’s interaction network (Fig. 9a) and was notably found to be differentially expressed between MBR and CON groups in males, but not in females. Furthermore, this gene was not included in the female MBR–CON module, suggesting *PPP1CC* may be a key gene overexpressed in the male MBR group and relevant to male-specific differences in individual response to trauma.

In females, the MBR–CON module was enriched for biological adhesion and extracellular space and significant hits among REACTOME pathways corroborated GO terms, involving cell junction organization, cell-cell communication, and hemostasis. This module was also enriched for the complement pathway in the hallmark collection and for TFTs of MEF2, HNF1, and SRF, implicating these transcription factors in putatively coordinating MBR-associated upregulation in females.

No modules were detected before SV adjustment in males, but the MBR–CON module was identified in unadjusted blood expression levels for both the female subset (M1: 157 genes; Fig. 7c) and full set (M2: 89 genes; Fig. 7d). In fact, the modules based on unadjusted data were more significantly enriched for shared GO terms and other top hits. This indicates that MBR upregulation of wound healing processes, particularly involving hemostasis/platelets, and regulation of body fluid levels are independent of MBR associations with other covariates, which were modeled as 9 SVs in blood. We also observed this module was more strongly enriched in females (MBR.F: NES = 3.7; CON.F: NES = − 3.44) than in males (MBR.M: NES = 1.66; CON.M: NES = − 2.2).

Interestingly, while upregulation in the MBR group is shared between sexes, the M2 module based on the full unadjusted blood expression data shows sex differences in the EBR group, such that female EBR is upregulated while male EBR is downregulated for this wound process-related module (Fig. 7d). In fact, the downregulation in the male EBR group has a greater effect than the male control group (EBR.M: NES = − 2.64; CON.M: NES = − 2.2). This suggests there may be a sex-specific EBR response to stress/trauma for the processes represented in this module. As this sexually dimorphic association is observed only in this module on the full, unadjusted data and not detected in sex-stratified SV-adjusted modules that highlight the contrast between MBR and CON groups, enrichment terms unique to this module may be most relevant for these sexually dimorphic EBR effects. The reactive oxygen species (ROS) pathway was identified as a top hit only in the full, unadjusted M2 module in gene set analyses, implicating response to ROS/oxidative stress as a high-priority pathway for sex differences in the EBR group.

In addition to these MBR-related findings, a module associated with downregulation in EBR and relative upregulation in unexposed CON group was identified in females (M2: 112 genes; Fig. 7b). This female-specific CON–EBR module was significantly enriched for GO terms related to immune and defense response, as well as coagulation, IL6-JAK-STAT3 signaling, and interferon gamma response in the hallmark gene set collection. These hallmark processes were also significantly associated with female EBR vs. MBR and EBR vs. CON contrasts in gene set enrichment analyses of differential expression results (Additional file 2 is available from the corresponding author upon request). Notably, interferon gamma (IFNγ) response was the top hit in gene set enrichment analyses of both the female EBR vs. MBR and EBR vs. CON comparisons, suggesting IFNγ may be a key player involved in PTSD-like response to trauma in females.

## Discussion

In this study, we investigated expression profiles of key PTSD-relevant tissues, namely blood, amygdala, and hippocampus, from a rat model of PTSD, to identify sex-specific and shared processes associated with individual differences in response to recent trauma exposure. By estimating cell proportions from brain expression profiles, we found higher proportions of hippocampal oligodendrocytes in the PTSD-like EBR group compared to the resilient MBR and unexposed CON group in both sexes, and this was supported by enrichment for oligodendrocyte markers in network analyses on unadjusted hippocampal data. While no group-related differences in cell proportions were detected in the amygdala, prominent sex differences were noted, with females showing significantly greater variance in distribution of relative cell proportions for astrocytes, neurons, oligodendrocytes, and oligodendrocyte progenitor cells. Cell proportion estimates and SVs were also used for data adjustment in network analyses to identify gene modules reflecting transcriptional activity, rather than coordinated expression reflecting a mix of cellular composition and transcriptional activity. By accounting for cell proportions and SVs, we were able to identify a number of shared and sex-specific gene expression modules reflecting group differences in transcriptional activity, in hippocampus, amygdala, and blood.

### Hippocampus

#### Group-based differences

Our finding of higher hippocampal oligodendrocyte proportions in the EBR group is consistent with reports from a recent study that used a metric based on magnetic resonance imaging (MRI) to estimate hippocampal myelination in male veterans with and without PTSD (due to combat trauma) [42]; veterans with PTSD had significantly more hippocampal myelin than trauma-exposed controls, and there was a positive correlation between hippocampal myelination estimates and PTSD symptom severity [42]. Relatedly, in rats, immobilization stress and corticosterone have been demonstrated to induce oligodendrogenesis in the dentate gyrus (DG) of adult hippocampus [43], suggesting stress induces hippocampal myelin formation. In our study, post hoc Dunn test for hippocampal oligodendrocytes showed a stronger effect size in the EBR vs. MBR comparison than the EBR vs. CON comparison and no significant difference between the MBR and CON groups, suggesting that differences in hippocampal oligodendrocyte proportions reflect PTSD-relevant differences in response to trauma (consistent with the study in humans) rather than differences due to trauma exposure (consistent with the study in rats).

Diffusion tensor imaging studies have endorsed significant alterations in white matter (WM) integrity, as measured by fractional anisotropy (FA), which are associated with trauma exposure, current PTSD diagnosis, current and lifetime PTSD symptom scores, PTSD status (e.g., remitted vs. persistent), and treatment outcome [44–47]. Both decrease [44, 47] and increase [45, 46] in FA were reported in different brain regions/tracts and contexts, with WM changes suggested to change over the course of PTSD. Moreover, investigation in oligodendrocyte precursor cell (OPC) culture demonstrated an interplay between proinflammatory cytokines and corticosteroids, such that IFNγ and TNFα impair survival and maturation of OPCs and co-administration of corticosteroids, most prominently dexamethasone, counters these deleterious effects [48]. Taken together, these studies support a model where dynamic, brain region-specific changes in white matter associated with stress/trauma exposure and PTSD pathophysiology reflect stress and neuroinflammation-dependent remodeling at the cellular/molecular level via dynamics in myelination and OPC survival, proliferation, and differentiation/maturation.

#### Sex differences by group

Findings from our hippocampal network analyses endorse group differences in transcriptional activity that fit into the framework of this model and the broader PTSD literature on hippocampus, which has consistently endorsed diminished neuronal and functional integrity [49]. Furthermore, our results suggest that there may be sex differences. Among the modules identified across hippocampal network analyses (i.e., full dataset vs. sex-stratified, SV-adjusted vs. cell estimate adjusted), we highlight a module associated with upregulation in the EBR group in each sex (discussion of male findings is presented in Additional file 1: Discussion) and one notable module significantly associated with upregulation in MBR and downregulation in EBR that was detected only in females

First, the female-specific MBR–EBR module was significantly enriched for extracellular structure organization, ECM receptor interaction, long-term synaptic potentiation, ensheathment of neurons, neurological system processes, and gliogenesis. When taken together, these enrichment results endorse sex-specific group differences in the ECM and associated signaling, which may be related to glial regulation of hippocampal synaptic plasticity, specifically long-term synaptic potentiation which has implications for learning and memory, via ensheathment of neurons. As oligodendrocyte proportions were found to be higher in the EBR than the MBR group, enrichment for gliogenesis and ensheathment of neurons may potentially reflect involvement of other glial cells, such as astrocytes that may provide non-myelinating ensheathment of neuronal synapses [50]. Since our cell proportion estimates only capture the major brain cell types, they are unable to inform changes in subpopulations of the major brain cell types. Given the diversity of astrocytes as a cell population, our results could indicate gliogenesis of relevant astrocyte subtypes (e.g., protoplasmic) or other glial cells that may not be captured by estimation of major brain cell types and imply participation of multiple glial cell types. Interestingly, this hippocampal female MBR–EBR module was also significantly enriched for genes with high-CpG-density promoters (HCPs) bearing histone H3K4me3 and H3K27me3 marks in brain, suggesting dynamic regulation of trauma-exposed group differences in female hippocampus via this epigenetic mechanism. In summary, findings from this module suggest differences in individual response to trauma in females may involve glial processes supporting synaptic connectivity in the hippocampus, putatively via astrocyte-synapse interactions. These processes, which are upregulated in the resilient MBR group and downregulated in the PTSD-like EBR group, may safeguard fear extinction learning in the MBR group and be implicated in impaired fear extinction learning in the EBR group.

Next, consistent evidence across three network analyses, in both the SV- and cell-adjusted full dataset and female subset of SV-adjusted data, endorsed increased expression of genes regulated by nuclear factor-κΒ (NFκΒ) in response to tumor necrosis factor-α (TNFα) and upregulation of genes after neuregulin-1 (NRG1) stimulation in the EBR group relative to the unexposed CON group in females, but not males. Stress has been shown to induce increased levels of proinflammatory cytokines, including TNFα, which activates NFκΒ, a key inflammatory transcription factor [51]. In the hippocampus, the TNFα signaling via NFκΒ pathway has been implicated in regulating synaptic plasticity and memory [52], and stress has been shown to activate NFκΒ signaling and decrease proliferation of neural stem cell-like cells in adult hippocampus [53]. Administration of an NFκΒ inhibitor has been demonstrated to block stress-induced inhibition of adult hippocampal neurogenesis, suggesting NFκΒ signaling may be a critical mediator of cellular (e.g., antineurogenic) consequences of stress that are linked to depressive-like behavior [53]. Relatedly, this module was also consistently enriched for genes upregulated after stimulation with NRG1. While this gene set was based on experiments conducted on a breast cancer cell line (MCF-7) [54] and caution should be exercised in interpreting implications in brain, NRG1 is generally involved in activation of proliferation, survival, and differentiation. Thus, upregulation of identified gene module members may reflect a general response to NRG1 that is not cell line specific. NRG1 is a member of the EGF family of receptor tyrosine kinase protein ligands with multiple isoforms and is known to be an important neurotrophic factor in the brain with key roles in neural development, synaptic plasticity, brain activity homeostasis, and neuroinflammation [55, 56]. Disruption of normal NRG/ERBB signaling has been implicated in impaired brain functioning and a number of psychiatric disorders [55], with the *NRG1* gene most notably implicated in schizophrenia [57, 58]. In hippocampus, NRG1 is suggested to regulate synapse development, particularly formation and maturation of inhibitory synapses, and has been shown to reduce expression of γ-aminobutyric acid (GABA) receptor α subunits in hippocampal slices [59].

#### Summary of transcriptional findings in hippocampal tissue

In summary, our results in hippocampus have detected distinct, putatively sex-specific, transcriptional activity characteristic of the PTSD-like EBR group and differential response to trauma in females involving glial processes supporting hippocampal synaptic connectivity that may be implicated in fear extinction learning. EBR in females was characterized by stress-induced upregulation of the TNFα signaling via NFκΒ pathway and disruption of NRG1/ERBB signaling, which may be involved in dysregulated synaptic plasticity and development in the hippocampus. Notably, these mechanisms may be implicated in the formation and maturation of inhibitory GABAergic synapses, with consequences for modulation of neuronal excitotoxicity. EBR in males was explicitly associated with increased regulation of GABAergic synaptic transmission and was also characterized by upregulated T-cell receptor signaling. Additionally, three transcription factors implicated in stress, inflammatory response, and T-cell functioning, namely GR, CEBPα, and LFA-1, were identified as candidates that may coordinate these processes.

### Amygdala

While no group differences were observed in brain cell subtype proportions, cell composition analyses revealed prominent sex differences in the distribution of amygdalar cell subtype proportions, such that females, regardless of group, showed significantly greater variance in distribution of relative cell proportions for astrocytes, neurons, oligodendrocytes, and oligodendrocyte progenitor cells, compared to males. The original Daskalakis et al. (2014) [20] study that deposited this dataset did not mention controlling for or tracking cycle or conducting ovariectomies, indicating the female rats in this dataset were free-cycling. Thus, the broader distribution of these cell proportions may potentially reflect sensitivity of these amygdalar cell types to gonadal steroid hormones.

Extensive sex differences are found at the cellular and physiological level in the amygdala, most notably in the posterodorsal medial amygdala (MePD), and are associated with organizational and activational effects of gonadal steroid hormones [60–66]; they are observed prior to puberty [60, 67, 68], become more extensive during puberty [69], and demonstrate steroid-dependent plasticity during adulthood [61–66]. In rat MePD, which is important for sex-specific behavior, studies have reported more neurons and astrocytes in males than in females, and additional sex differences in size and morphology of cell bodies and processes, which also differ by laterality [62, 67, 68, 70]. While not specific to amygdala, sexual dimorphism of oligodendrocyte response to sex hormones has been reported in cell culture with females consistently showing greater sensitivity and responsiveness to hormones than males [71]. To our knowledge, brain region-specific differences have not yet been investigated.

Network analyses in amygdala detected both shared and sex-specific gene modules. The resilient MBR group in both sexes was characterized by increased expression of ECM-relevant genes (e.g., cytoskeletal proteins) and upregulation of ECM organization/remodeling, with collagen fibril organization specifically implicated. Additionally, enrichment for epithelial mesenchymal transition implicated enhanced migratory capacity and stem cell-like phenotype with processes in the likeness of wound healing and tissue repair in response to stress-induced neuroinflammation [72]. The second shared module was significantly associated with downregulation in EBR for both sexes and relative upregulation in MBR females; thus, our results endorse this module to reflect group differences in response to trauma in females, but only supports downregulation of this module to be characteristic of the EBR group in males.

With respect to the PTSD-like EBR group, this shared module was associated with downregulation of GPCR signaling, synaptic signaling/transmission, GABAβ receptor activation, and exploration behavior, as well as decreased regulation of insulin secretion by glucagon-like peptide 1, protein kinase A (PKA) signaling, and cAMP metabolic process. GPCR signaling, PKA signaling, and cAMP metabolic processes are ubiquitous and involved in signaling transduction; in the cell, cAMP targets PKA, which serves as the principal effector mechanism for GPCRs linked to adenylate cyclase [73]. The cAMP/PKA signaling pathway is involved in the regulation of glucose homeostasis [74] and relevant to the decreased regulation of insulin secretion by glucagon-like peptide 1. Additionally, the cAMP/PKA signaling pathway plays an essential, evolutionarily conserved role in the mediation of neural processing of threat-related stimuli (i.e., fear learning), consolidation of fear memory, and fear-related behavioral response in the amygdala [73]. Targeted activation of cAMP/PKA signaling in the lateral amygdala has been shown to increase neuronal excitability and lead to generalized fear [75], while targeted inhibition of cAMP/PKA activity in the lateral amygdala immediately after fear conditioning impaired fear memory retention [76]. Additionally, stress modulates fear memories; β-adrenoceptor-mediated activation of the cAMP/PKA pathway has been shown to enhance fear memory consolidation, and glucocorticoids (GCs) interact with the noradrenergic signaling pathway to modulate this activation in the basolateral amygdala (BLA) [73]. Targeted inhibition of either β-adrenoceptor or cAMP/PKA in the BLA before or immediately post inhibitory avoidance training has been shown to block memory consolidation and administering a GR antagonist before this training was able to block the noradrenergic-dependent enhancement of memory retention [77]. Fear learning and memory is also linked to other anxiety-related behavioral responses, including exploration behavior, which was also significantly downregulated in this module. Thus, dysregulation of this pathway in the amygdala is directly relevant for pathogenesis of PTSD.

The male EBR module was characterized by decreased expression of genes regulated by NFκΒ in response to TNFα, which is the opposite direction of effect seen in hippocampus for the female EBR group. Both the hippocampus and amygdala are stress-sensitive brain regions where NFκΒ plays a critical role in memory and is necessary for fear memory consolidation and reconsolidation [78–80]. However, the exact region- and sex-specific differences in downstream effects of TNFα/NFκΒ signaling are unknown and require further investigation. Additional discussion of the male-specific findings may be found in Additional file 1: Discussion. In sum, blunted GC response, disrupted inhibitory modulation of amygdalar circuitry, diminished NPY levels, and dysregulation of genes downstream of TNFα/NFκΒ signaling are implicated in the male EBR group.

More PTSD-relevant modules were identified in females; two modules (based on cell and SV-adjusted female subset) were associated with upregulation in MBR and downregulation in EBR, with the cell-adjusted module largely overlapping with the shared module identified in the full, cell-adjusted dataset. This module was significantly enriched for the previously identified gene sets and was also enriched for additional terms. Notably, significant enrichment for inhibition of insulin secretion by adrenaline/noradrenaline and behavior/associative learning elaborated a potential role for nor/adrenergic signaling in glucose homeostasis and strengthened the implications in fear learning and anxiety-related behavioral response. Additionally, downregulation of dopamine receptor signaling, specifically for dopamine D_2_ receptor (*Drd2*), was significantly associated with this module. Dopaminergic signaling plays an important modulatory role in fear learning/memory and anxiety. D2 receptors in the amygdala are endorsed to have context-specific effects and an important role in regulating fear/anxiety responses [81, 82], with putatively differential functions in the central versus the lateral amygdala [83], which receives key dopaminergic innervations from the ventral tegmental area [84] and mediates dopaminergic gating of LTP induction necessary for fear conditioning [85]. Notably, dopaminergic innervation of the amygdala is suggested to be more responsive to stress exposure than other limbic brain regions [86], implying dopamine signaling in the amygdala may be important in stress response. Thus, differential dopaminergic and GABAergic signaling in the amygdala may underlie important group differences in the modulation of fear learning and memory between resilient and PTSD-like females.

The second female-specific module strongly endorses *Srf* as a key driver for trauma-exposed group differences in the female amygdala (see Additional file 1: Discussion for more discussion of these findings). Additionally, the module was indicated for differential expression of genes regulated by TNFα signaling via NFκΒ. This is in line with our results for amygdala in the male EBR group, which was also characterized by downregulation of this gene set. However, opposing direction of effect was observed for this gene set in the female EBR group across amygdala and hippocampus. Surprisingly, inspection of overlapping gene module members revealed there were more genes overlapping between the hippocampal and amygdalar modules with opposite directions of effect in females than between the male and female amygdalar modules with same direction of effect, suggesting putative sex and brain region-specific regulation in response to TNFα signaling via NFκΒ. The different direction of effect between amygdala and hippocampus in females parallels previous studies demonstrating stress to increase synaptic plasticity, BDNF levels, and induce dendritic hypertrophy in the amygdala, while having the opposite effect in hippocampus and medial prefrontal cortex [87–90]. This is also endorsed by the one amygdalar module, detected in females, associated with upregulation in EBR relative to the unexposed CON group; this female EBR module was characterized by upregulation of genes involved in the ECM, stem cell/NPC proliferation, positive regulation of neuron differentiation, and positive regulation of dendritic spine development. Thus, TNFα signaling via NFκΒ may be involved in the regulation of stress-mediated synaptic/structural plasticity in both the hippocampus and amygdala, but have region-specific effects. Additionally, there may be more nuanced sex differences in genes/processes altered by TNFα signaling via NFκΒ.

#### Summary of findings in amygdala tissue

In summary, our results in amygdala support fundamental sex differences in cell composition that may be significantly influenced by sex hormones. While a number of sex-specific modules are endorsed, some group-relevant modules were shared across sexes. The resilient MBR group was characterized by upregulation of ECM organization/remodeling, illustrating processes similar to wound healing and tissue repair in response to stress-induced neuroinflammation in the amygdala. The PTSD-like EBR group was characterized by disruption of cAMP/PKA signaling, GABAβ receptor activation, and κ-OR signaling in the amygdala, which lead to dysregulation of synaptic signaling, impaired fear learning/memory, and anxiety-related behavioral response. Additionally, TNFα signaling via NFκΒ was indicated to play a major role in mediating stress-induced synaptic/structural plasticity in both sexes, but implicated for sex-specific downstream effects. In males, EBR was also characterized by the decreased expression of neuropeptide hormones, including those use to define classes of BLA interneurons, suggesting an additional male-specific mechanism involved in the disruption of excitatory/inhibitory balance. Notably, downregulation of NPY may be directly linked to behavioral response and was only detected in male, but not female, amygdala. On the other hand, in females, disruption of dopamine D_2_ receptor signaling and the Srf transcription factor were suggested to be key drivers for trauma-exposed group differences in the amygdala, where they are purported to shape differences in excitatory/inhibitory balance, differentially activate IEGs and differentially mediate cytoskeletal dynamics in neuronal processes. Together, they may drive fundamental group differences in experience-dependent modulation of structural plasticity, and this has implications for long-term fear learning and memory. Additionally, we noted these PTSD-relevant modules in amygdala were significantly enriched for genes with HCPs bearing H3K4/27me3 in brain and were stronger in sex-stratified modules, except in the female module characterized by transcriptional regulation via Srf. Thus, epigenetic regulation of transcriptional activity is endorsed in the amygdala, in both sexes.

### Blood

A module characterizing MBR in blood was identified for each sex. In both sexes, this module was associated with general upregulation of wound healing processes, particularly platelet activation/hemostasis, and increased regulation of body fluid levels. These processes were also identified using unadjusted expression levels, suggesting they are independent of MBR associations with other covariates modeled by SVs. Interestingly, the unadjusted module also revealed that while upregulation of this module is shared across sexes in the MBR group, stress response in represented processes is sexually dimorphic in the EBR group, such that the module is upregulated in the female EBR and downregulated in male EBR group.

The findings from this module for males directly mirror gene network analyses conducted in humans (using blood) [91, 92]. A prospective cohort study in US Marines pre- and post- deployment identified a module after combat trauma exposure (post-deployment) associated with PTSD resiliency signatures and an upregulation of genes involved in hemostasis and wound responsiveness [91]. A more recent mega-analysis combined five independent blood transcriptome datasets and investigated three PTSD case-trauma-exposed control groups, stratified by type of trauma exposure and sex, specifically men with combat trauma, men with interpersonal (IP) trauma, and women with IP trauma. They identified a wound-healing and coagulation module downregulated in men with PTSD and combat trauma (relative to trauma-exposed control males with combat trauma). This module was not relevant to women, which is in line with our results that indicated upregulation in both the female MBR and EBR groups. Interestingly, the module also was not identified in men with IP trauma either, suggesting associated processes may be trauma-exposure specific.

Furthermore, our results suggest response to ROS/oxidative stress may be a candidate pathway to target in the study of sex differences in PTSD. Sex differences in oxidative stress have previously been implicated in the study of cardiovascular diseases, which also have a sex/gender bias in disease prevalence [93] and are often co-morbid with PTSD [6, 7]. Sex differences in response to oxidative stress have also been reported in the brain, notably in the hippocampus, in response to ethanol withdrawal [94] and prenatal stress [95]. Moreover, these sex differences appear to be evolutionarily conserved with reports of sex-specific adaptation to oxidative stress even reported in fruit flies [96].

The sex-stratified MBR–CON modules also endorsed some processes to be sex-specific. Vascular smooth muscle contraction is supported to be a process more relevant in males with *PPP1CC* identified as a candidate hub gene upregulated in the male MBR group. Upregulation of the complement pathway and is supported to be more relevant in females with HNF1 and SRF found to be candidate transcription factors involved in coordinating this MBR module in females. Additionally, a module identified only in females suggested female EBR may be characterized by downregulation of immune and defense responses, which may involve impairment of coagulation, IL6-JAK-STAT3 signaling, and interferon gamma (IFNγ) response, with IFNγ particularly implicated as a key player involved in PTSD-like response to stress in females. This is in line with a study that reported several sex-specific effects of IFNγ treatment on monoaminergic activity in key limbic regions and also demonstrated an IFNγ by stress (acute restraint stress) interaction to increase corticosterone levels in plasma, with larger effects in males [97].

#### Summary of findings in blood

In summary, we found wound healing processes and hemostasis to be upregulated in the resilient MBR group across sexes, but disrupted in a sexually dimorphic manner in the EBR group. These findings, particularly in males, are consistent with the PTSD literature. Response to oxidative stress and IFNγ is endorsed as candidate processes to target in the study of sex differences in PTSD.

### Cross-tissue and integrated remarks

In general, upregulated ECM remodeling and wound healing processes appears to be a common MBR response to stress/trauma that is observed across tissues and shared between sexes. Systemic stress hormones, particularly glucocorticoids (GCs), act in both blood and brain. The hippocampus and amygdala are highly plastic and stress-sensitive limbic regions central for learning and memory associated with strong emotions–prominently fear memories, which have direct implications for PTSD symptoms. Stress-induced TNFα/NFκΒ signaling, which plays a key role in the regulation of synaptic plasticity and fear learning/memory, is suggested to be a key pathway differentially disrupted in the EBR group in both brain regions. Although the specific downstream disruption is region-specific, a common theme of excitatory/inhibitory imbalance, involving disruption of GABAergic signaling, is advocated. In females, differential NRG1/ERBB signaling is implicated with region-specific effects in hippocampus and amygdala.

Another notable commonality across hippocampus and amygdala was upregulation of ECM-regulated genes and ECM organization/remodeling in the resilient MBR group, in response to stress-induced neuroinflammation. This is akin to a wound healing/tissue repair response in the brain and parallels significant upregulation of wound healing processes characteristic of MBR in blood. Notably upregulation of ECM/wound healing is shared across sexes in both amygdala and blood, of both MBR groups. While upregulation of ECM was identified in a female-specific hippocampal module, no MBR-upregulated module was detected for males among adjusted, sex-stratified network analyses, and there was generally more difficulty identifying PTSD-relevant hippocampal modules in males than females. This challenge may stem from the loss of two male hippocampal samples during QC, which resulted in four male hippocampal samples in the EBR and MBR groups (8 samples total). Thus, sex-specificity needs to be revisited with a larger sample size, especially in hippocampus.

As mentioned, a major limitation of this study was sample size, with some sex-stratified groups in brain having only four samples, namely male EBR and male MBR groups in hippocampus and female MBR group in amygdala. By identifying co-expressed gene modules using an unsupervised network analysis approach and converging evidence across analyses based on sex-stratified and full datasets adjusted for SV and cell estimates (in brain), we were able to identify the gene modules that were most consistently supported across analyses. Additionally, further validation and investigation is required for cell estimation approaches, particularly in rats, due to lack of reference datasets.

### Perspectives and significance

Despite these study limitations, our findings suggest both shared and sex-specific mechanisms underlying individual differences associated with vulnerability and resilience to trauma in blood and two key limbic areas, namely hippocampus and amygdala. By disentangling cell composition from transcriptional changes, we found higher proportions of hippocampal oligodendrocytes in the EBR group for both sexes and also identified modules for transcriptional activity associated with group differences (i.e., response to trauma) in the hippocampus that appeared to be sex-specific. By contrast, we found prominent sex differences, but no group differences, in amygdalar cell composition, and both shared and sex-specific modules representing PTSD-relevant transcriptional activity in the amygdala. Across amygdala and hippocampus, both sex-specific and shared processes were relevant to an overarching framework for EBR implicating disrupted TNFα/NFκΒ signaling and excitatory/inhibitory imbalance in dysregulated synaptic/structural plasticity with important implications for fear learning and memory. Our main finding in peripheral blood was consistent with the human literature and identified wound healing processes and hemostasis to be upregulated in the MBR group across sexes, but disrupted in a sexually dimorphic manner in the EBR group. Unlike the varied characterization of the EBR group, characterization of MBR across blood, amygdala, and hippocampus suggests a common theme of upregulated wound healing and ECM remodeling shared between sexes. In all, we identified differential oligodendrocyte proportions in hippocampus between PTSD-like EBR and resilient MBR, and identified processes and pathways that characterize the EBR and MBR-associated transcriptional changes across hippocampus, amygdala, and blood. The sex-specific mechanisms involved in EBR may contribute to the pronounced disparity in risk for PTSD, with women much more likely to develop PTSD.

## Supplementary information

**Additional file 1.** Supplementary methods with details on QC, data processing, and gene annotation steps.

**Additional file 2.** Gene co-expression network analysis reports and gene set analysis results for modules of interest.

## Data Availability

Additional file 2 is available from the corresponding author upon request. The original datasets supporting the conclusions of this article are available in the Gene Expression Omnibus repository, https://www.ncbi.nlm.nih.gov/geo/query/acc.cgi?acc=GSE60280; https://www.ncbi.nlm.nih.gov/geo/query/acc.cgi?acc=GSE60302; https://www.ncbi.nlm.nih.gov/geo/query/acc.cgi?acc=GSE60303.
