# Supplementary material for: Sex-specific and shared expression profiles of vulnerability and resilience to trauma in brain and blood
Source: Biol Sex Differ. 2020 Mar 30;11:13. doi: 10.1186/s13293-020-00288-6 (PMC7106761; doi:10.1186/s13293-020-00288-6)
Supplement: Supplementary file 1 — Additional file 1. Supplementary methods with details on QC, data processing, and gene annotation steps. [file 13293_2020_288_MOESM1_ESM.zip › Kim_Additional_File_One.docx]

**Additional File 1**

**Methods**

**Quality Control and Data Processing**

Expression datasets for each tissue were *normexp* background-corrected, quantile normalized, and log_2_ transformed, separately using *neqc* in the *limma* package [1, 2]. For background correction, observed intensities were fit to a normal+exponential convolution model, estimating model parameters using detection p-value to infer mean and variance of negative control probes. Probes were filtered for low expression in each tissue dataset and annotation quality (out of 22,523 total probes). Retained probes had a detection p-value < 0.01 in at least 10% of subjects, consistent chromosome mapping between annotation sources, and mapping to human orthologs. For 1:1 gene:probe mapping, genes mapped to multiple probes were represented by the probe with highest mean expression levels across samples in each tissue dataset. After data processing, each tissue dataset retained 4,901 expressed genes (represented by 5,065 probes) in blood, 6,653 genes (represented by 6,869 probes) in hippocampus, and 6,811 genes (represented by 7,029 probes) in amygdala.

**Annotation**

Probe annotations were based on platform, organism, and transcriptome level annotations packaged in Bioconductor (version 3.7)[3, 4] databases. Re-annotated gene symbols (based on re-mapping of Illumina Rat v1 probe sequences with ReMOAT[5]; *illuminaRatv1.db*) were used to retrieve updated NCBI Entrez Gene IDs (April 2018; *org.Rn.eg.db*). Entrez Gene IDs were used to map genes to UCSC rn5 genome coordinates (*TxDb.Rnorvegicus.UCSC.rn5.refGene*; April 2018) and predicted human orthologs in the Molecular Signatures Database (MSigDB v6.2.1, released July 2018; *msigdbr*). For multi-mapping genes, human ortholog is chosen within MSigDB based on support by the largest number of databases (eggNOG, Ensembl Compara, HGNC, HomoloGene, Inparanoid, NCBI Gene Orthology, OMA, OrthoDB, OrthoMCL, Panther, PhylomeDB, TreeFam and ZFIN).

**Cell Composition Analysis in Brain Tissue**

Shapiro-Wilk test was used to assess normality and Levene’s test (using median as center; modified Brown-Forsythe) was used to compare equality of variance among groups. Since not all cell subtype estimates had normal distributions (by population), Mann-Whitney U test was used to compare mean ranks of cell estimates for initial comparisons by trauma-exposed group (EBR vs MBR) and sex, across cell subtypes, when equality of variance was met. Two-sample Kolmogorov-Smirov (KS) test was used to compare distribution of non-normal cell estimates when variances were unequal between groups. For cell subtypes of interest that met modeling assumptions, one-way analysis of covariance (ANCOVA) was used to test main effect of each variable (EBR vs MBR). Kruskal-Wallis and post-hoc Dunn tests were used for group comparisons including trauma un-exposed controls. A threshold of 0.05 was used for p-values and p-values were adjusted for multiple comparisons using Holm’s method[6] in cell composition analyses.

**Differential Expression and Gene Set Enrichment Analyses in Blood**

Association between group and gene expression due to transcriptional changes was assessed by fitting a linear regression model of gene expression as a function of sex-stratified group while accounting for SVs as covariates in the model. Sex-stratified contrasts were applied to the model fit to investigate EBR vs. MBR, EBR vs. CON, and MBR vs. CON differences. The linear model and sex-stratified contrasts were fitted using an empirical Bayes approach in *limma* to compute moderated t-statistics and log_2_ fold change (logFC)[1, 7] for each probe in each comparison, to determine differential expression (DE) for each sex-stratified comparison (FDR-adjusted p-value < 0.05).

Pre-ranked gene set enrichment analysis (GSEA)[8] was conducted for each sex-stratified comparison using a sorted vector of logFC as input. The *fgsea* algorithm for fast gene set enrichment analysis[9] was implemented via *clusterProfiler*[10] for gene sets in the hallmark gene set collection from the Molecular Signatures Database (MSigDB v6.2, released July 2018)[8, 11] that had a minimum of 10 and maximum of 500 genes. The p-value of the enrichment score (representing the degree to which a gene set is over-represented at the top or bottom of the ranked logFC list) was calculated by permutation test (1000 permutations) and adjusted for multiple hypothesis testing using the Benjamini-Hochberg (BH) method[12] (*adj p* < 0.05).

**Gene Co-expression Network Analysis**

The β power parameter selection was conducted after variance-based filtering of expression data, using default settings. For construction of signed co-expression networks, the weighted network adjacency matrix was calculated by raising the co-expression similarity measure to the selected β, and biweight midcorrelation (bicor) was used as the co-expression measure, unless otherwise noted. For module detection, genes were assigned to modules using the dynamic tree cutting method[13] on branches of the hierarchical clustering dendrogram of the signed topological overlap dissimilarity matrix (1-TOM). Modules with eigengene correlations > 0.8 were merged and minimum module size was set to 30 genes. Group differences for activity of identified gene modules (i.e., upregulation or downregulation; induced/repressed) were determined via *CEMiTool* using the *fgsea* algorithm[9], treating genes in co-expressed modules as gene sets and the z-score normalized expression of samples within each group as rankings on the analysis.

**Results**

**Sex-stratified network analyses of SV-adjusted data in hippocampal tissue**

Sex-stratified network analyses on SV-adjusted data detected a second module in the female subset, which was associated with upregulation in MBR and downregulation in the EBR group (M1: 170 genes; Figure S1B). This female-specific MBR – EBR module was significantly enriched for neurological system process, proteinaceous extracellular matrix (ECM), extracellular structure organization, ensheathment of neurons, and long-term synaptic potentiation among GO terms (Figure S2). ECM relevant terms were corroborated by significant enrichment for ECM receptor interaction among KEGG pathway gene sets (*adj p* = 0.009, 6/31genes). Interestingly, the top hit in the C2 CGP collection was for genes with high-CpG density promoters (HCP) bearing histone H3K4me3 and H3K27me3 in brain (adj p = 0.0012, 28/387 genes; *MEISSNER_BRAIN_HCP_WITH_H3K4ME3_AND_H3K27ME3*). In embryonic stem cells, genes containing HCPs enriched with both H3K4me3 and H3K27me3 histone marks (associated with transcription initiation and repression, respectively) were found to be highly regulated developmental genes[14], and this histone state is itself dynamic and correlated with DNA methylation levels[15]. Thus, enrichment for this gene set suggests the module is dynamically regulated by this epigenetic mechanism.

Network analyses on the male subset of SV-adjusted hippocampal expression data most notably identified one module associated with upregulation in the EBR group (M2: 101 genes; NES 1.75; Figure S1C). This EBR-related module was significantly enriched for regulation of GABAergic synaptic transmission, the TCR pathway, G alpha I signaling events, G-protein coupled receptor (GPCR) signaling, and transcription factor targets (TFTs) for GR, CEBP, and LFA1 (Table S2).

**Sex-stratified network analyses of SV-adjusted data in amygdalar tissue**

Network analyses based on the full SV-adjusted dataset did not identify any notable PTSD-relevant modules, but sex-stratified network analyses on SV-adjusted data identified one large module, using Spearman correlation as the co-expression measure, in the male amygdalar subset and three modules in the female subset (Figure S3). In females, module M3 (108 genes), associated with downregulation in EBR and upregulation in MBR, was significantly enriched for GO terms related to TF activity (RNAPII core promoter, proximal region sequence-specific binding), particularly for transcriptional activator activity, and muscle tissue/structure development. Analyses in other gene set collections identified top enrichment for TNFα signaling via NFκΒ (*adj p* = 1.5e-08, Hallmark), four gene sets associated with the SRF transcription factor (C3 TFT), and genes downregulated in neurons after NPAS4 knockdown by RNAi (*adj p* = 1.34e-06).

In males, the identified PTSD-relevant module was associated with downregulation in the EBR and relative upregulation in the CON group (M1: 453 genes). The top GO term enriched in this module was GPCR signaling pathway, which was corroborated by significant terms from the REACTOME gene set collection and agreed with the top GO term associated with module M3 from cell-adjusted amygdalar data, suggesting this pathway may be generally downregulated in the EBR group from both sexes. Other GO terms significantly enriched in this male M1 module were response to organic cyclic compound/lipid/(steroid) hormone/nitrogen compound. The module was also significantly enriched for neuroactive ligand receptor interaction (KEGG, *adj p* = 0.016), TNFα signaling via NFκΒ (Hallmark, *adj p* = 0.002), hypoxia (Hallmark, *adj p* = 0.03), and *MEISSNER_BRAIN_HCP_WITH_H3K4ME3_AND_H3K27ME3* (C2 CGP, top hit, *adj p* = 5.06e-09).

**Discussion**

**Sex differences by group in hippocampal tissue**

In males, the module upregulated in the EBR group was associated with genes involved in the regulation of GABAergic synaptic transmission as well as those involved in the T-cell receptor (TCR) signaling pathway. In addition to its inhibitory role in synaptic transmission, GABAergic signaling plays a parallel role in the immune system[16] and has been shown to inhibit adaptive inflammatory response to myelin proteins in an animal model of multiple sclerosis (i.e., experimental autoimmune encephalomyelitis [EAE])[17]. Similarly, relevant to this male EBR-associated module, GABAergic signaling negatively regulates T cell-mediated immunity by its modulatory effect on T-cell proliferation and cytokine production[18], and has been shown to inhibit TCR-mediated T-cell cycle progression *in vitro*[19]. Thus, GABAergic signaling may putatively play a dual role in the regulation of both synaptic transmission and T-cell programming in this male EBR module.

The male EBR module was also significantly enriched for GPCR signaling and for genes with 3’UTR containing motif matching annotations for GR (glucocorticoid receptor, *NR3C1*), CEBPα (CCAAT/enhancer binding protein), and LFA-1 (lymphocyte function-associated antigen-1). While GPCR signaling is ubiquitous, in the context of tissue and other significant enrichment hits for this module, fits a narrative of G-protein mediated signaling promoting initial adhesion of inflammatory T-cells on blood brain barrier endothelium (BBB) for transendothelial migration to hippocampus, with a putative role for LFA-1 in this process, under neuroinflammatory conditions[20]. In parallel, stimulation of LFA-1 also modulates transcriptional programming for a number of key pathways, including activation of T lymphocyte differentiation into effector subsets Th1, Th17, and induced regulatory T cells, for tuning of T-cell functioning[21]; this modulation of transcriptional programming is the function directly implicated by our enrichment results. In a similar vein, CEBPα is involved in inflammatory response and regulate expression of many pro-inflammatory genes shown to have increased expression after injury to aged brain[22]. Finally, GR is an important mediator of stress response that is implicated in stress-related psychiatric disorders. Glucocorticoids in the hippocampus, distinct from their effect in hypothalamus, have been shown to play an important role in the development of hypothalamus-pituitary adrenal (HPA) axis hyperactivity[23]. Of note, acute stress has been shown to increase interaction of GRs with their genomic targets in the hippocampus, in a gene-dependent manner[24] and chronic stress has been implicated in dysfunction of GABAergic interneurons in the hippocampus[25].

**Sex differences by group in amygdalar tissue**

The second female-specific module strongly endorses *Srf* as a key driver for trauma-exposed group differences in the female amygdala. In the adult brain, the *Srf* transcription factor activates immediate early genes (IEGs) and mediates cytoskeletal dynamics at neuronal processes[26]. Enrichment results support differential regulation of both these effects, which suggests fundamental group differences in experience-dependent modulation of synaptic plasticity involving structural changes[27]; in amygdala, this has implications for long-term fear learning and memory. Notably, the top hit in the C2 CGP collection indicates enrichment for genes downregulated in neurons after *Npas4* knockdown, suggesting downstream effects of *Srf* disruption in the female EBR group may involve disruption of *Npas4* activation. In the amygdala, *Npas4* expression was found to be selectively induced under conditions of associative learning and is suggested to be important for fear memory consolidation/reconsolidation[28]. It has been demonstrated to regulate activity-dependent development of GABAergic inhibitory synapses via transcriptional regulation of the activity-dependent genes directly involved[29], which fits the theme of disrupted excitatory-inhibitory balance in the amygdala.

In males, one module was significantly associated with downregulation in EBR and relative upregulation in CON, suggesting that this module highlights EBR response to trauma exposure. Accordingly, a number of the terms significantly enriched with the module were related to response a molecular factor, notably to lipid, hormone, and steroid hormone, implicating enrichment for genes downregulated in response to glucocorticoids (GCs). This male-specific EBR module was also significantly associated with downregulation of neuropeptide hormone activity, which included decreased expression of cholecystokinin (*Ckk*), neuropeptide Y (*Npy*), and vasoactive intestinal peptide (*Vip*). These neuropeptides define classes of BLA interneurons that provide feedforward and feedback inhibition to projection neurons and other interneurons locally and are likely to be involved in higher-order contextualized behavior such as fear extinction[30, 31]. Thus, downregulation of these neuropeptides may be indicative of a decrease in these inhibitory amygdalar interneurons and disruption of excitatory/inhibitory balance.

Of these neuropeptides, NPY has been studied the most in relation to stress-related psychiatric disorders, as its release is induced by stress and has anxiolytic properties[32]. Notably, a study of NPY using the same predator-scent stress (PSS) paradigm found lower NPY levels in the amygdala of the male EBR group compared to MBR or CON and showed administration of NPY one hour post-exposure to significantly reduce prevalence rates of EBR and trauma-cue freezing responses compared to controls[33]. This suggests 1) downregulation of NPY is a robust characteristic of EBR directly linked to the behavioral response, and 2) NPY may serve not only as a diagnostic marker but also as a treatment candidate that can prevent development of PTSD-like symptoms and promote resilience. Interestingly, low NPY expression may be a predisposing risk factor. A human genetic imaging study identified an *NPY* haplotype predictive of *NPY* mRNA expression in postmortem brain and lymphoblast and plasma NPY levels; using functional (fMRI and PET) imaging, they found low NPY haplotype individuals exhibited greater amygdalar reactivity in response to threat-related facial expression, had lower activation of pain/stress-induced μ-opioid system activation, and reported more negative emotional experiences during a painful stressor compared to high NPY haplotype individuals[34].

The male EBR module was also characterized by decreased expression of genes regulated NFκΒ in response to TNFα, which is the opposite direction of effect seen in hippocampus for the female EBR group. Both the hippocampus and amygdala are stress-sensitive brain regions where NFκΒ plays a critical role in memory and is necessary for fear memory consolidation and reconsolidation[35-37]. However, the exact region- and sex- specific differences in downstream effects of TNFα/NFκΒ signaling are unknown and require further investigation. In sum, blunted GC response, disrupted inhibitory modulation of amygdalar circuitry, diminished NPY levels, and dysregulation of genes downstream of TNFα/NFκΒ signaling are implicated in the male EBR group.

**References**

1. Ritchie ME, Phipson B, Wu D, Hu Y, Law CW, Shi W, et al. limma powers differential expression analyses for RNA-sequencing and microarray studies. Nucleic Acids Res. 2015;43(7):e47.

2. Shi W, Oshlack A, Smyth GK. Optimizing the noise versus bias trade-off for Illumina whole genome expression BeadChips. Nucleic Acids Res. 2010;38(22):e204.

3. Huber W, Carey VJ, Gentleman R, Anders S, Carlson M, Carvalho BS, et al. Orchestrating high-throughput genomic analysis with Bioconductor. Nat Methods. 2015;12(2):115-21.

4. Gentleman RC, Carey VJ, Bates DM, Bolstad B, Dettling M, Dudoit S, et al. Bioconductor: open software development for computational biology and bioinformatics. Genome Biol. 2004;5(10):R80.

5. Barbosa-Morais NL, Dunning MJ, Samarajiwa SA, Darot JF, Ritchie ME, Lynch AG, et al. A re-annotation pipeline for Illumina BeadArrays: improving the interpretation of gene expression data. Nucleic Acids Res. 2010;38(3):e17.

6. Holm S. A Simple Sequentially Rejective Multiple Test Procedure. Scandinavian Journal of Statistics. 1979;6(2):65-70.

7. Smyth GK. limma: Linear Models for Microarray Data. 2005. [398-420].

8. Subramanian A, Tamayo P, Mootha VK, Mukherjee S, Ebert BL, Gillette MA, et al. Gene set enrichment analysis: a knowledge-based approach for interpreting genome-wide expression profiles. Proc Natl Acad Sci U S A. 2005;102(43):15545-50.

9. Sergushichev AA. An algorithm for fast preranked gene set enrichment analysis using cumulative statistic calculation. bioRxiv preprint. 2016.

10. Yu G, Wang LG, Han Y, He QY. clusterProfiler: an R package for comparing biological themes among gene clusters. OMICS. 2012;16(5):284-7.

11. Liberzon A, Subramanian A, Pinchback R, Thorvaldsdottir H, Tamayo P, Mesirov JP. Molecular signatures database (MSigDB) 3.0. Bioinformatics. 2011;27(12):1739-40.

12. Benjamini Y, Hochberg Y. Controlling the False Discovery Rate - a Practical and Powerful Approach to Multiple Testing. J Roy Stat Soc B Met. 1995;57(1):289-300.

13. Langfelder P, Zhang B, Horvath S. Defining clusters from a hierarchical cluster tree: the Dynamic Tree Cut package for R. Bioinformatics. 2008;24(5):719-20.

14. Bernstein BE, Mikkelsen TS, Xie X, Kamal M, Huebert DJ, Cuff J, et al. A bivalent chromatin structure marks key developmental genes in embryonic stem cells. Cell. 2006;125(2):315-26.

15. Meissner A, Mikkelsen TS, Gu H, Wernig M, Hanna J, Sivachenko A, et al. Genome-scale DNA methylation maps of pluripotent and differentiated cells. Nature. 2008;454(7205):766-70.

16. Wu C, Qin X, Du H, Li N, Ren W, Peng Y. The immunological function of GABAergic system. Frontiers In Bioscience. 2017;22:1162-72.

17. Bhat R, Axtell R, Mitra A, Miranda M, Lock C, Tsien RW, et al. Inhibitory role for GABA in autoimmune inflammation. Proc Natl Acad Sci U S A. 2010;107(6):2580-5.

18. Dionisio L, Jose De Rosa M, Bouzat C, Esandi Mdel C. An intrinsic GABAergic system in human lymphocytes. Neuropharmacology. 2011;60(2-3):513-9.

19. Tian J, Lu Y, Zhang H, Chau CH, Dang HN, Kaufman DL. Gamma-aminobutyric acid inhibits T cell autoimmunity and the development of inflammatory responses in a mouse type 1 diabetes model. J Immunol. 2004;173(8):5298-304.

20. Engelhardt B. Molecular mechanisms involved in T cell migration across the blood-brain barrier. J Neural Transm (Vienna). 2006;113(4):477-85.

21. Verma NK, Kelleher D. Not Just an Adhesion Molecule: LFA-1 Contact Tunes the T Lymphocyte Program. J Immunol. 2017;199(4):1213-21.

22. Sandhir R, Berman NE. Age-dependent response of CCAAT/enhancer binding proteins following traumatic brain injury in mice. Neurochem Int. 2010;56(1):188-93.

23. Zhu LJ, Liu MY, Li H, Liu X, Chen C, Han Z, et al. The different roles of glucocorticoids in the hippocampus and hypothalamus in chronic stress-induced HPA axis hyperactivity. PLoS One. 2014;9(5):e97689.

24. Mifsud KR, Reul JM. Acute stress enhances heterodimerization and binding of corticosteroid receptors at glucocorticoid target genes in the hippocampus. Proc Natl Acad Sci U S A. 2016;113(40):11336-41.

25. Banasr M, Lepack A, Fee C, Duric V, Maldonado-Aviles J, DiLeone R, et al. Characterization of GABAergic marker expression in the chronic unpredictable stress model of depression. Chronic Stress (Thousand Oaks). 2017;1.

26. Knoll B, Nordheim A. Functional versatility of transcription factors in the nervous system: the SRF paradigm. Trends Neurosci. 2009;32(8):432-42.

27. Ramanan N, Shen Y, Sarsfield S, Lemberger T, Schutz G, Linden DJ, et al. SRF mediates activity-induced gene expression and synaptic plasticity but not neuronal viability. Nat Neurosci. 2005;8(6):759-67.

28. Ploski JE, Monsey MS, Nguyen T, DiLeone RJ, Schafe GE. The neuronal PAS domain protein 4 (Npas4) is required for new and reactivated fear memories. PLoS One. 2011;6(8):e23760.

29. Lin Y, Bloodgood BL, Hauser JL, Lapan AD, Koon AC, Kim TK, et al. Activity-dependent regulation of inhibitory synapse development by Npas4. Nature. 2008;455(7217):1198-204.

30. Krabbe S, Grundemann J, Luthi A. Amygdala Inhibitory Circuits Regulate Associative Fear Conditioning. Biol Psychiatry. 2018;83(10):800-9.

31. Babaev O, Piletti Chatain C, Krueger-Burg D. Inhibition in the amygdala anxiety circuitry. Exp Mol Med. 2018;50(4):18.

32. Enman NM, Sabban EL, McGonigle P, Van Bockstaele EJ. Targeting the Neuropeptide Y System in Stress-related Psychiatric Disorders. Neurobiol Stress. 2015;1:33-43.

33. Cohen H, Liu T, Kozlovsky N, Kaplan Z, Zohar J, Mathe AA. The neuropeptide Y (NPY)-ergic system is associated with behavioral resilience to stress exposure in an animal model of post-traumatic stress disorder. Neuropsychopharmacology. 2012;37(2):350-63.

34. Zhou Z, Zhu G, Hariri AR, Enoch MA, Scott D, Sinha R, et al. Genetic variation in human NPY expression affects stress response and emotion. Nature. 2008;452(7190):997-1001.

35. de la Fuente V, Federman N, Zalcman G, Salles A, Freudenthal R, Romano A. NF-kappaB transcription factor role in consolidation and reconsolidation of persistent memories. Front Mol Neurosci. 2015;8:50.

36. Yeh SH, Lin CH, Lee CF, Gean PW. A requirement of nuclear factor-kappaB activation in fear-potentiated startle. J Biol Chem. 2002;277(48):46720-9.

37. Si J, Yang J, Xue L, Yang C, Luo Y, Shi H, et al. Activation of NF-kappaB in basolateral amygdala is required for memory reconsolidation in auditory fear conditioning. PLoS One. 2012;7(9):e43973.

**Table S1:** Enrichment for M2 module associated with EBR vs CON in female subset of SV-adjusted hippocampal expression data

|  | **Gene Ratio** | | | **Bg Ratio** | **adj p** | **geneID** | |
| --- | --- | --- | --- | --- | --- | --- | --- |
| C2: CGP (top hit) |  | | |  |  |  |  |
| NAGASHIMA_NRG1_SIGNALING_UP | 18/144 | | | 99/6477 | 5.3e-09 | *SGK1/EGR1/JUNB/TOB2/PER1/ARC/DUSP8/NFIL3/SIK1/*  *NR4A2/EGR4/IER2/FOS/IER5/KDM6B/GADD45B/KLF2/ZFP36* | |
| NAGASHIMA_EGF_SIGNALING_  UP | 10/144 | | | 36/6477 | 2.7e-06 | *EGR1/JUNB/ARC/SIK1/NR4A2/EGR4/IER2/FOS/KDM6B/ZFP36* | |
| H | |  |  | |  |  |  |
| TNFA_SIGNALING_VIA_NFKB | 17/52 | | | 94/2230 | 2.5e-10 | *SGK1/EGR1/NFKBIA/JUNB/PER1/NFIL3/SIK1/CFLAR/NR4A2/KLF4/IER2/FOS/IER5/KDM6B/GADD45B/KLF2/ZFP36* | |
| C5: GO BP (top hits) |  | | |  |  |  |  |
| CELLULAR_RESPONSE_TO_  ORGANIC_CYCLIC_COMPOUND | 18/132 | | | 216/5807 | 0.0025 | *APLP1/SGK1/EGR1/PDE2A/NTS/NFKBIA/RAE1/IRF3/SIRT2/*  *ARG1/CDKN1B/NR4A2/KLF4/HCN2/BMP7/KLF2/KCNJ11*  */RAMP3* | |
| CELLULAR_RESPONSE_TO_  ENDOGENOUS_STIMULUS | 26/132 | | | 466/5807 | 0.0055 | *APLP1/SGK1/EGR1/PDE2A/NTS/WNT4/JUNB/MYO5A/ACVR2B/SIRT2/CDH13/ARG1/SIK1/PIK3R2/CACNA1H/NR4A2/KLF4/HCN2/RRAGD/FOS/BMP7/ENPP1/KLF2/KCNJ11/ASNS/RAMP3* | |

*Note:* p-values adjusted with Benjamini-Hochberg method.

Consider gene sets with a minimum of 10 and maximum of 500 genes. Universe of background genes is based on overlap of genes expressed in hippocampus (n = 6,489) with gene set collection.

First number in Gene Ratio refers to number of genes overlapping between term/gene set and module (i.e., gene count), while second number refers to number of genes overlapping between gene set collection and the module.

First number in Background Ratio (Bg Ratio) refers to universe of expressed genes in the gene set, while second number refers to universe of expressed genes in the gene set collection.

geneID lists genes in gene module that overlap with genes in gene set/term.

All gene set collections (blue rows) were from the Molecular Signatures Database (MSigDB v6.2, released July 2018).

C2: CGP = chemical and genetic perturbations sub-collection in curated gene set collection; 3,433 gene sets.

H = hallmark gene set collection; 50 gene sets.

C5: GO BP = biological process sub-collection in GO gene set collection; 4,436 gene sets.

**Table S2:** Enrichment for M2 module associated with EBR in male subset of SV-adjusted hippocampal expression data

|  | **Gene Ratio** | | | **Bg Ratio** | **adj p** | **geneID** | |
| --- | --- | --- | --- | --- | --- | --- | --- |
| C2: CP REACTOME | |  |  | |  |  |  |
| G_ALPHA_I_SIGNALLING_EVENTS | 7/42 | | | 69/2433 | 0.021 | *CXCL12/RGS4/SST/PENK/PDYN/CNR1/RGS8* | |
| GPCR_DOWNSTREAM_SIGNALING | 10/42 | | | 178/2433 | 0.04 | *CXCL12/PRKCE/NGEF/RGS4/SST/PENK/VIP/PDYN/CNR1/*  *RGS8* | |
| SIGNALING_BY_GPCR | 11/42 | | | 216/2433 | 0.04 | *CXCL12/PRKCE/NGEF/RGS4/SST/WNT4/PENK/VIP/PDYN/*  *CNR1/RGS8* | |
| C3 TFT |  | | |  |  |  | |
| GR_Q6 | 8/82 | | | 106/5191 | 0.041 | *TYRO3/WNT4/KLF5/DLX1/NNAT/CNTN4/NCAM1/LRRTM3* | |
| CEBP_Q2_01 | 8/82 | | | 107/5191 | 0.041 | *STMN1/TNNC2/KLF5/DLX1/CALML4/PTEN/EFEMP1/LRRTM3* | |
| LFA1_Q6 | 8/82 | | | 107/5191 | 0.041 | *WNT4/ANXA4/LCAT/DLX1/PDYN/ACVR1C/GSS/TMEM132E* | |
| C5: GO |  | | |  |  |  |  |
| REGULATION_OF_SYNAPTIC_  TRANSMISSION_GABAERGIC | 5/96 | | | 20/6137 | 0.02 | *PRKCE/HAP1/PTEN/PLCL2/CNR1* | |

*Note:* p-values adjusted with Benjamini-Hochberg method.

Consider gene sets with a minimum of 10 and maximum of 500 genes. Universe of background genes is based on overlap of genes expressed in hippocampus (n = 6,489) with gene set collection. First number in Gene Ratio refers to number of genes overlapping between term/gene set and module (i.e., gene count), while second number refers to number of genes overlapping between gene set collection and the module. First number in Background Ratio (Bg Ratio) refers to universe of expressed genes in the gene set, while second number refers to universe of expressed genes in the gene set collection. geneID lists genes in gene module that overlap with genes in gene set/term.

All gene set collections (blue rows) were from the Molecular Signatures Database (MSigDB v6.2, released July 2018).

C2: CGP = chemical and genetic perturbations sub-collection in curated gene set collection; 3,433 gene sets.

C2: CP REACTOME = REACTOME gene sets in canonical pathways sub-collection in curated gene set collection; 674 gene sets.

C3 TFT = transcription factor targets sub-collection in motif gene set collection; 615 gene sets. Significant gene sets suggest module enrichment for genes having at least one occurrence of transcription factor binding sites for V$GR_Q6, V$CEBP_Q2_01, and V$LFA1_Q6 (v7.4 TRANSFAC), in the regions spanning up to 4 kb around their transcription start sites.

C5: GO = GO gene set collection; 5,917 gene sets total.

**Figure Legends**

**Figure S1.** Module enrichment plot for PTSD-relevant modules identified in sex-stratified network analyses of hippocampus. In the SV-adjusted female subset: (A) Module M2 consists of 144 genes and is associated with upregulation in EBR and relative downregulation in CON; (B) Module M1 consists of 170 genes and is associated with upregulation in MBR and downregulation in EBR. In the SV-adjusted male subset: (C) Module M2 consists of 101 genes and is associated with upregulation in EBR. The size and intensity of the circles correspond to the normalized enrichment score (NES) for the module in each sex-stratified group (normalized by the number of genes in the module).

**Figure S2.** Gene-concept network plot for top GO terms enriched in female-specific MBR – EBR module derived from female subset of SV-adjusted hippocampal expression data (M1: 170 genes). Edges show associations between GO terms based on overlapping module genes. Size of circles, representing GO terms, reflect gene count.

**Figure S3.** Module enrichment plot for modules identified in sex-stratified network analyses of SV-adjusted amygdalar expression levels in (A) females and (B) males. The size and intensity of the circles correspond to the normalized enrichment score (NES) for the module in each class (normalized by the number of genes in the module).
