# Supplementary figures and images for: Sex-specific and shared expression profiles of vulnerability and resilience to trauma in brain and blood
Source: Biol Sex Differ. 2020 Mar 30;11:13. doi: 10.1186/s13293-020-00288-6 (PMC7106761; doi:10.1186/s13293-020-00288-6)

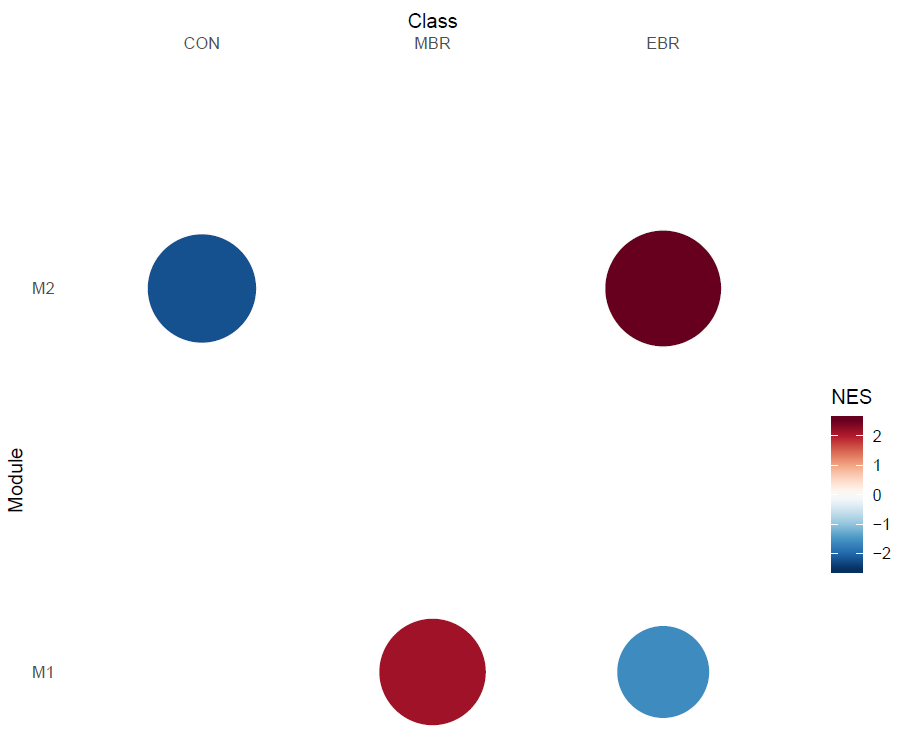

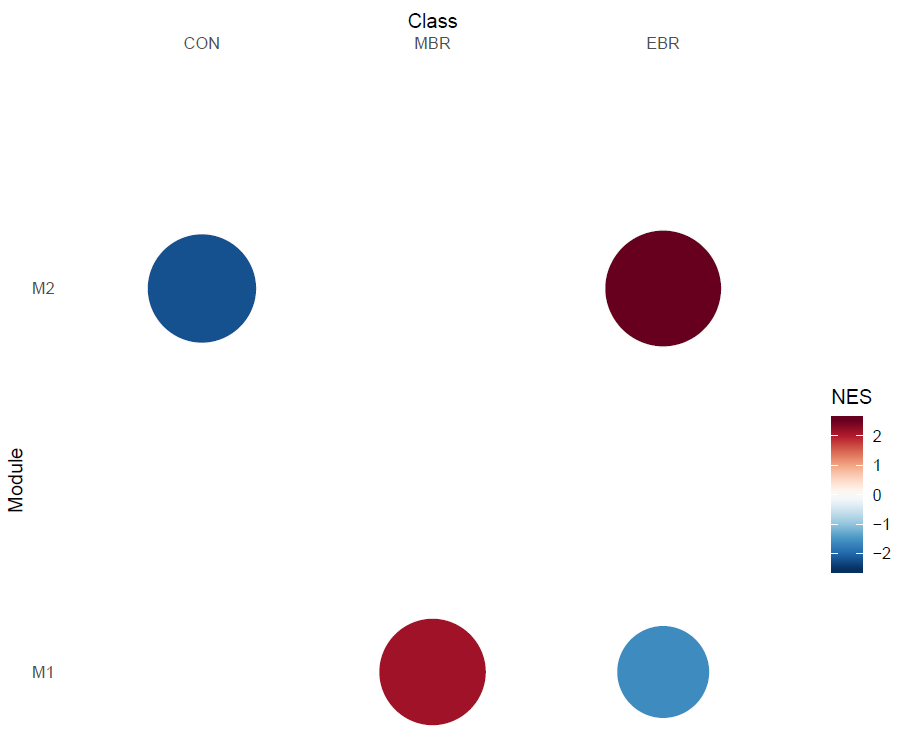


**A**


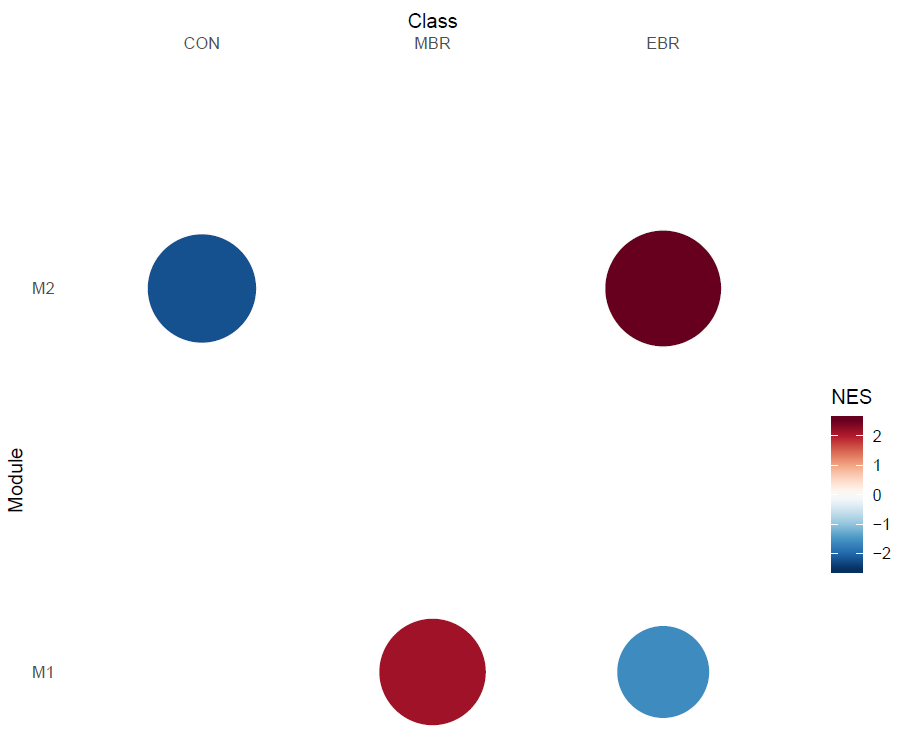

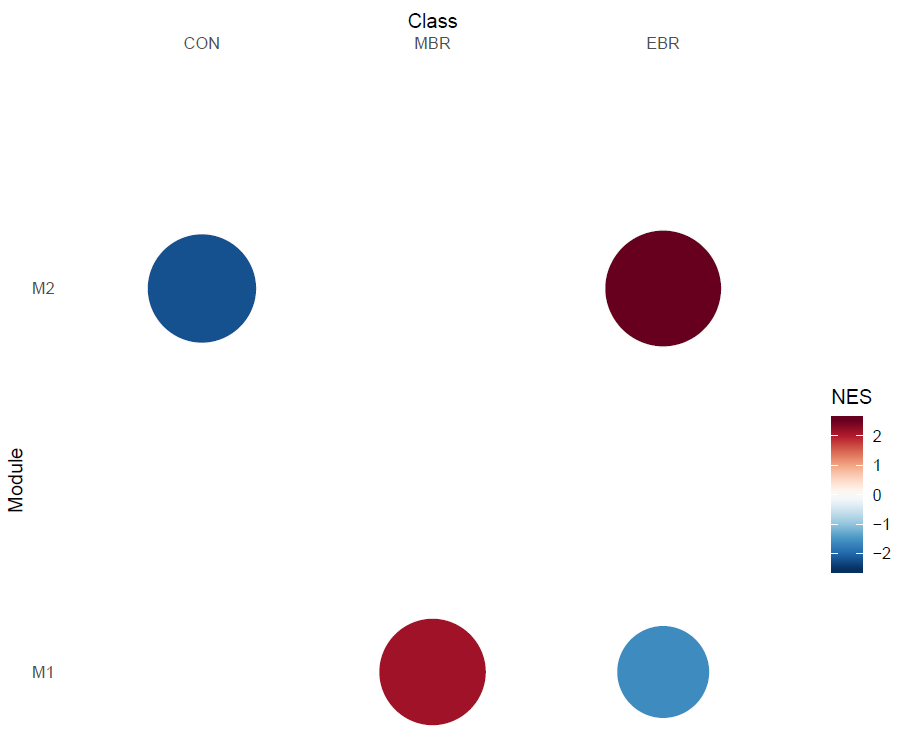

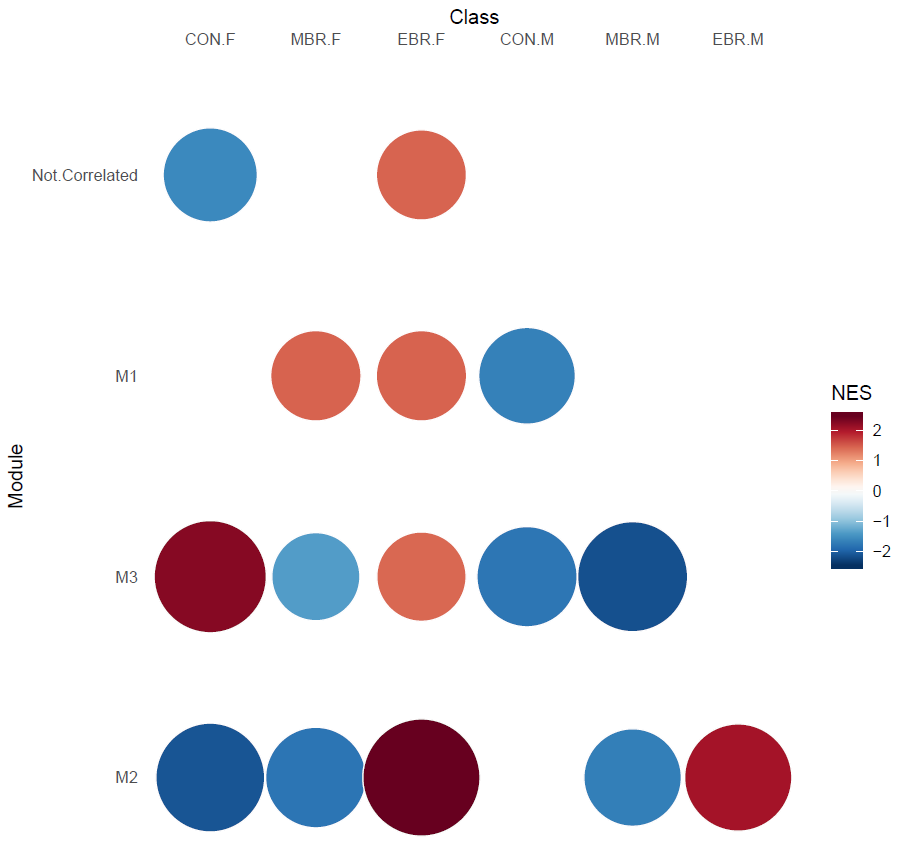


**B**


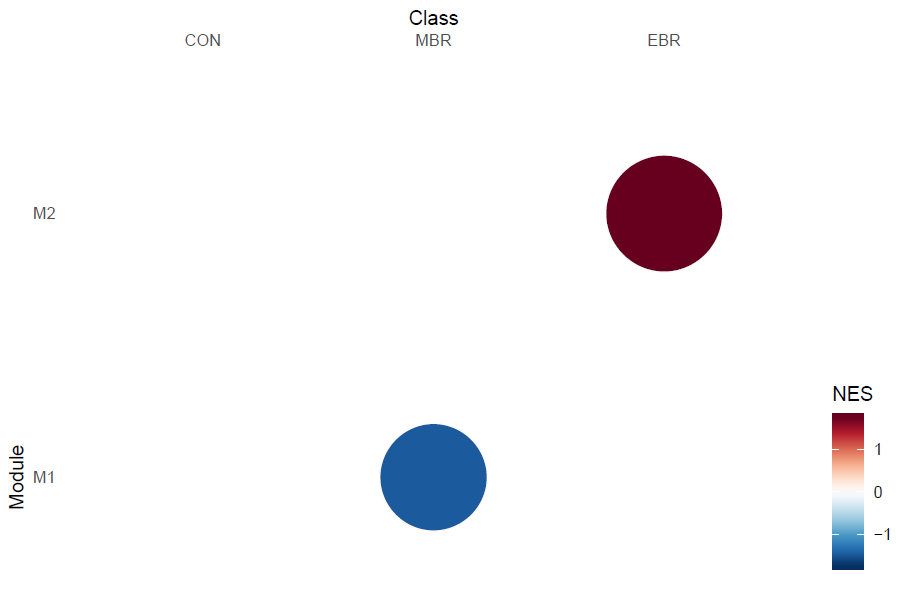

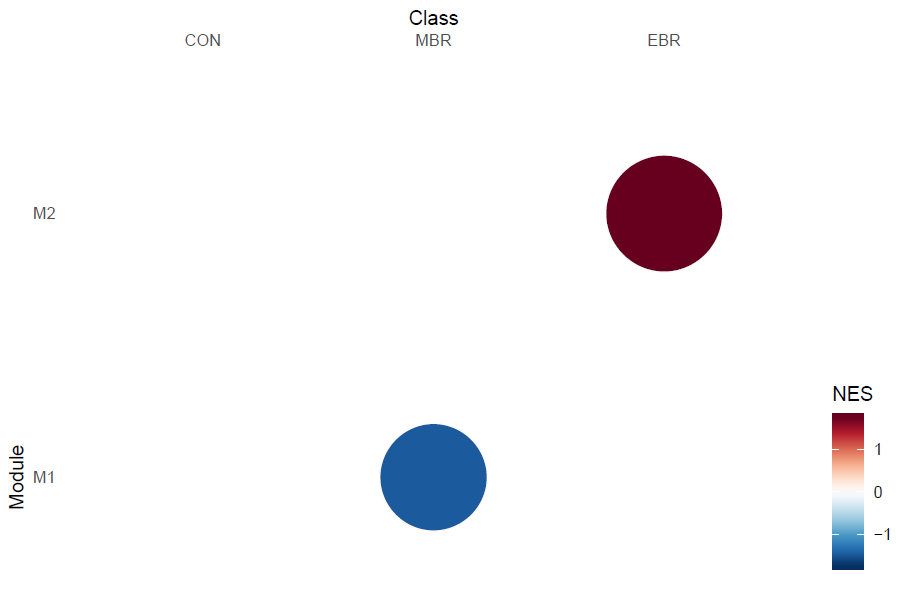


**C**


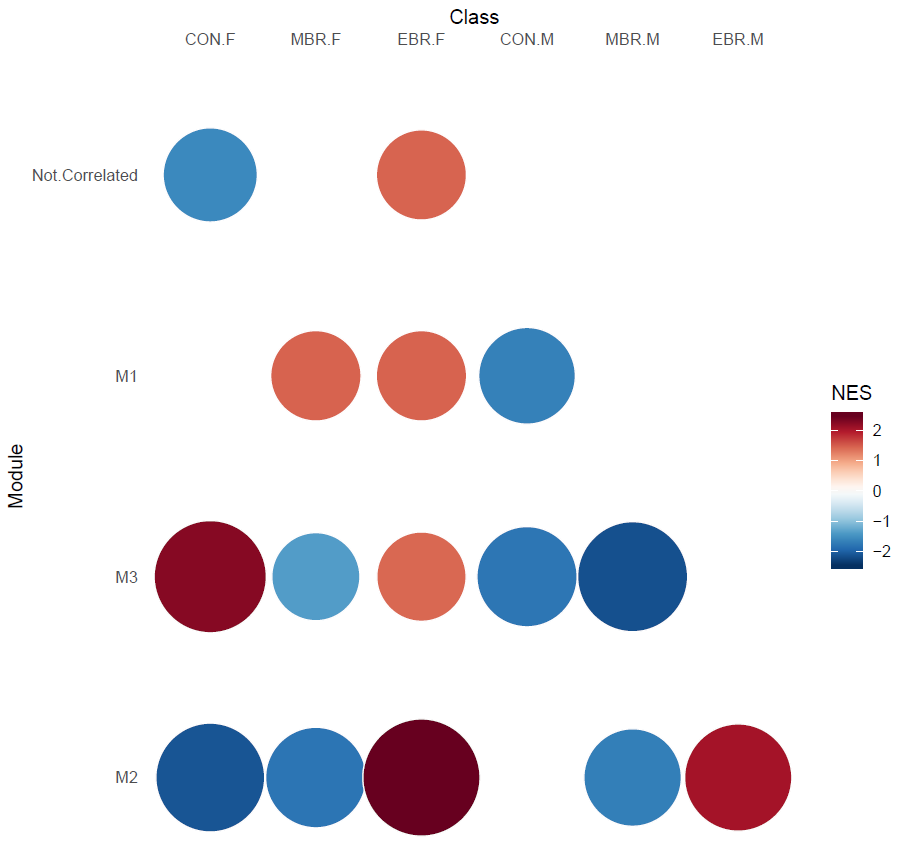

Supplement: Supplementary file 1 — Additional file 1. Supplementary methods with details on QC, data processing, and gene annotation steps. [file 13293_2020_288_MOESM1_ESM.zip › Figure S1.docx]

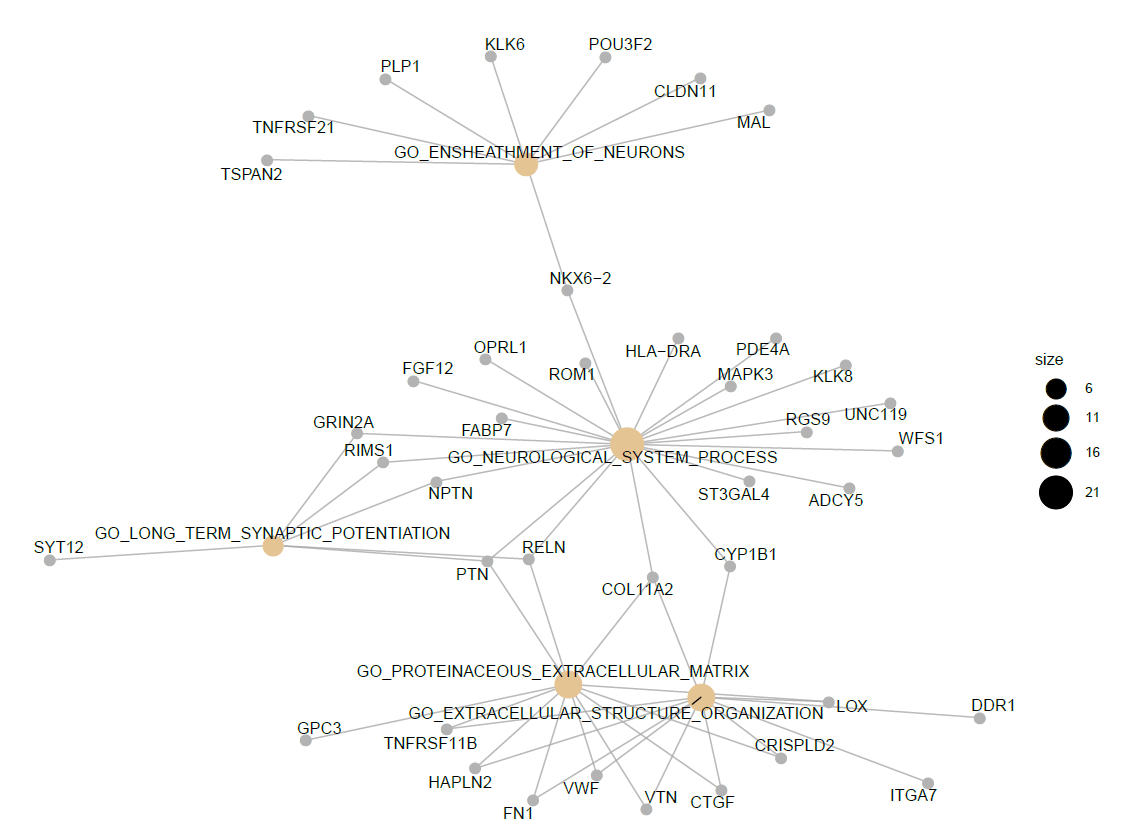

Supplement: Supplementary file 1 — Additional file 1. Supplementary methods with details on QC, data processing, and gene annotation steps. [file 13293_2020_288_MOESM1_ESM.zip › Figure S2.docx]

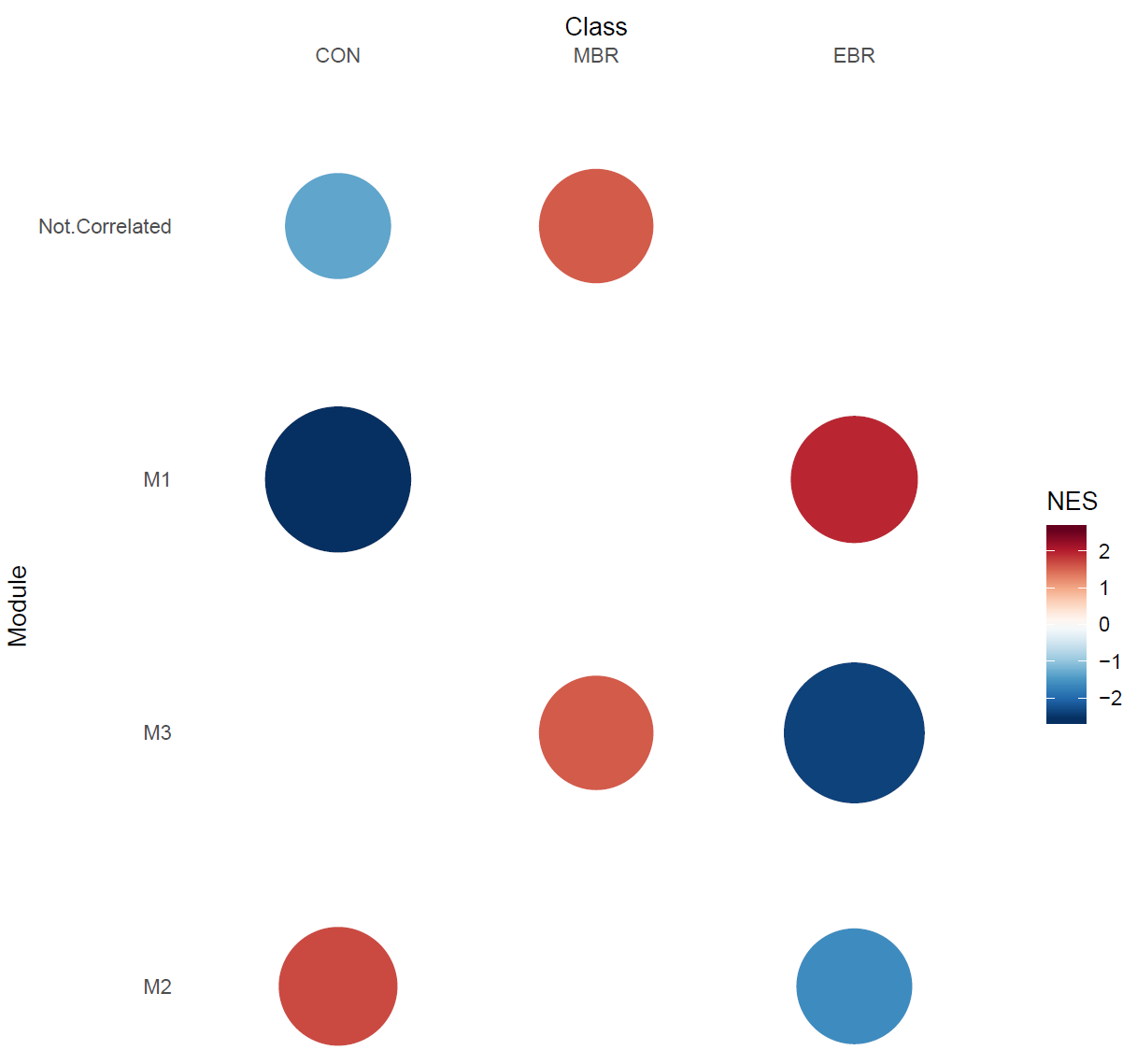

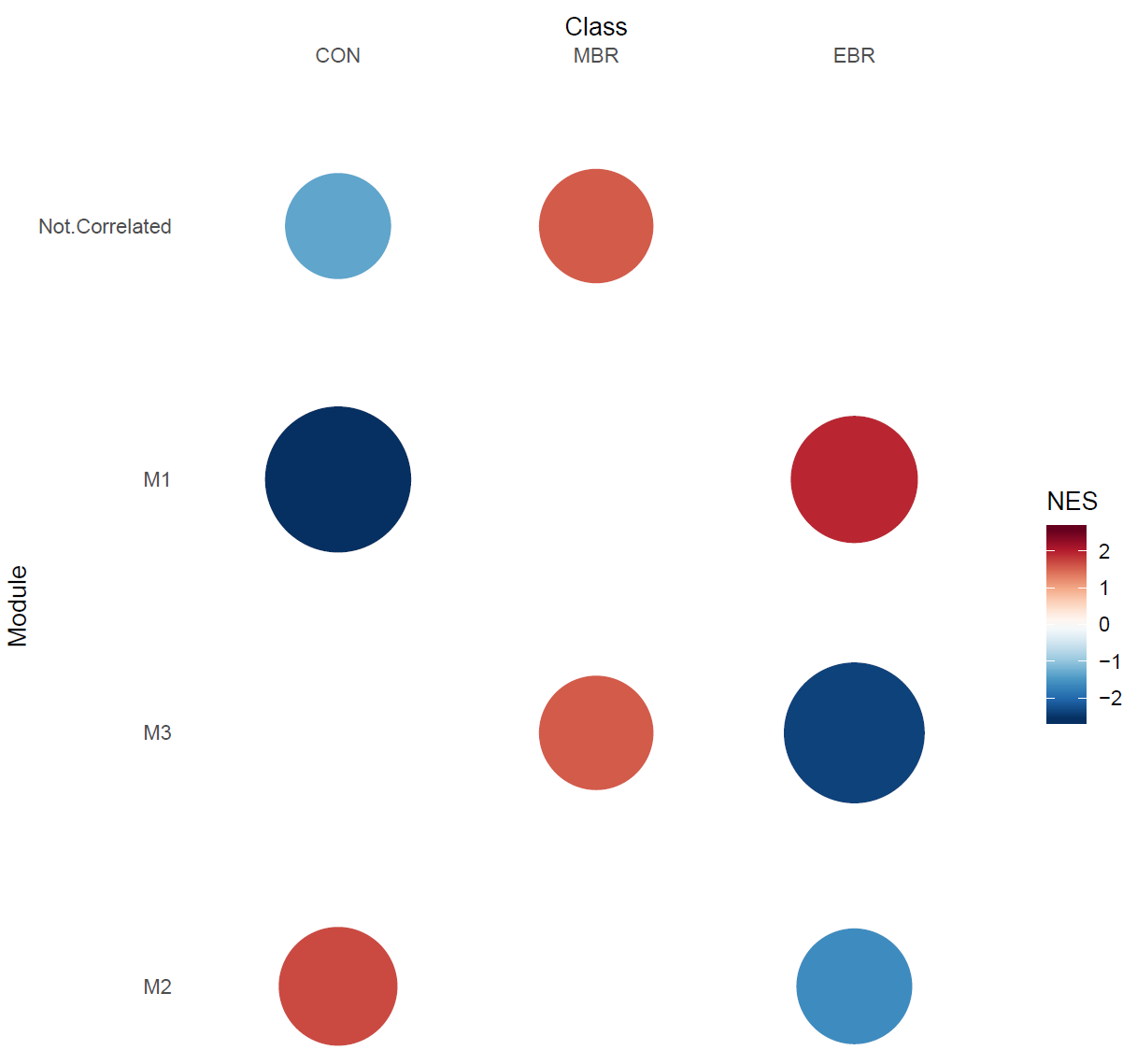

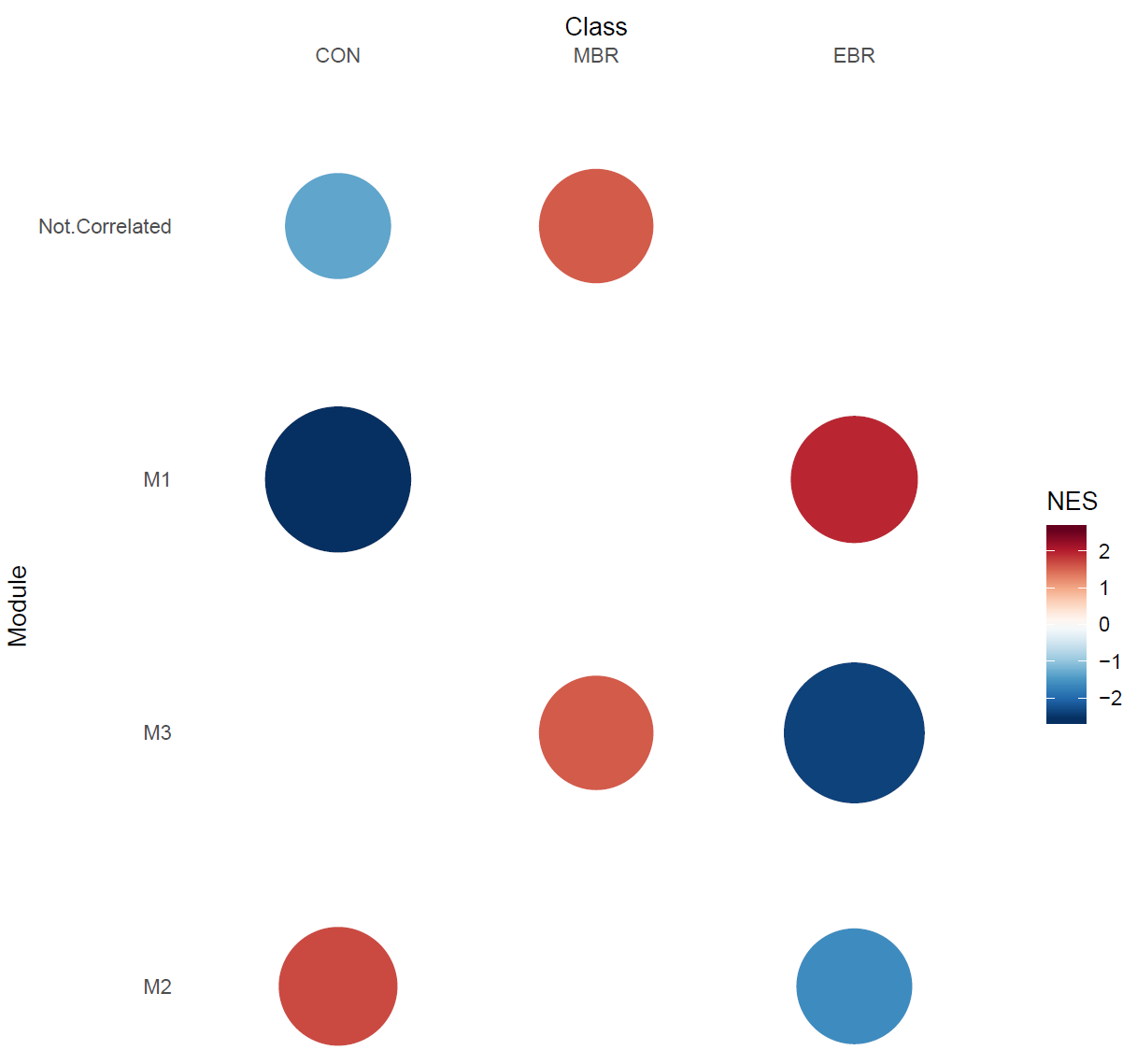

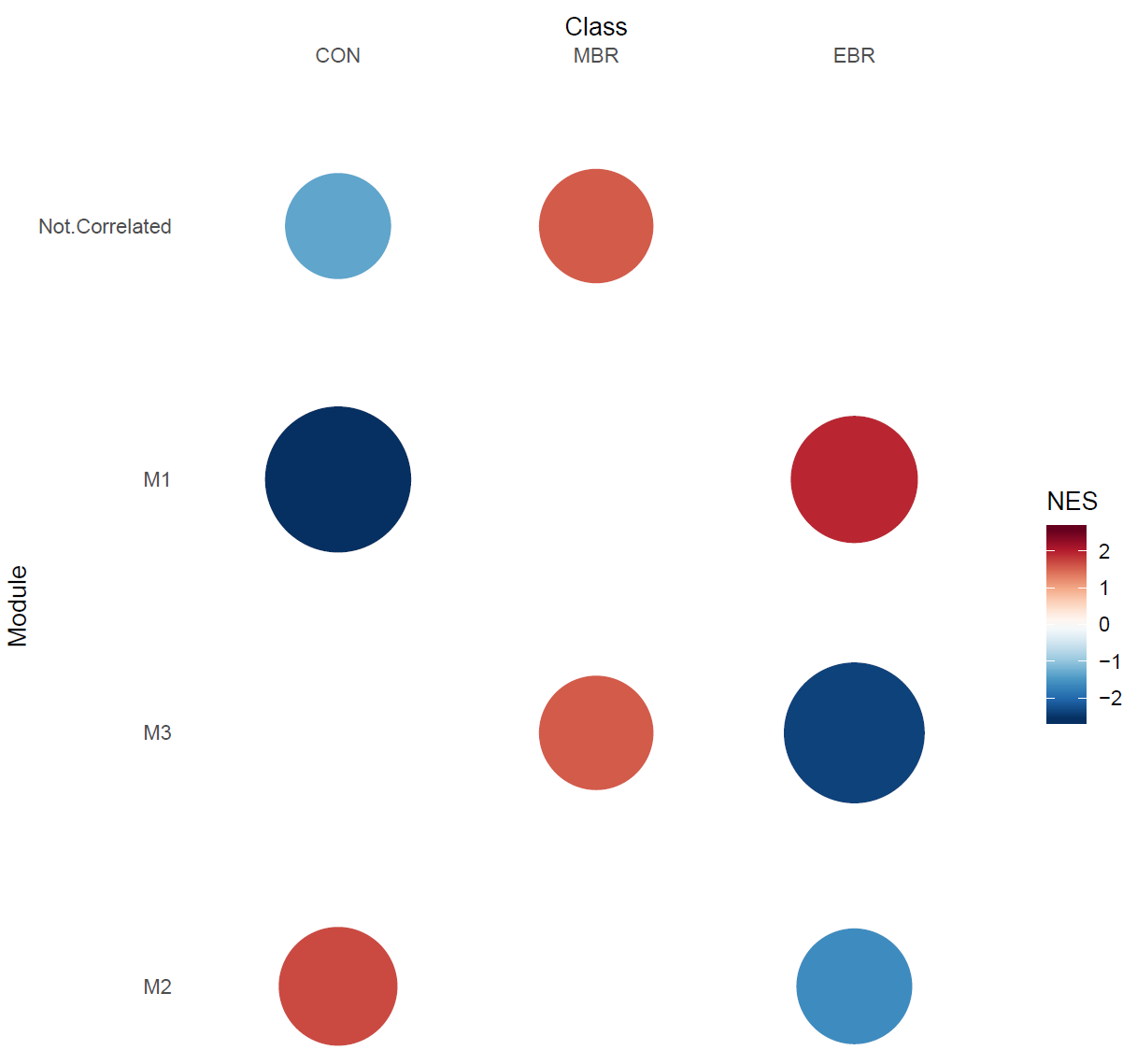

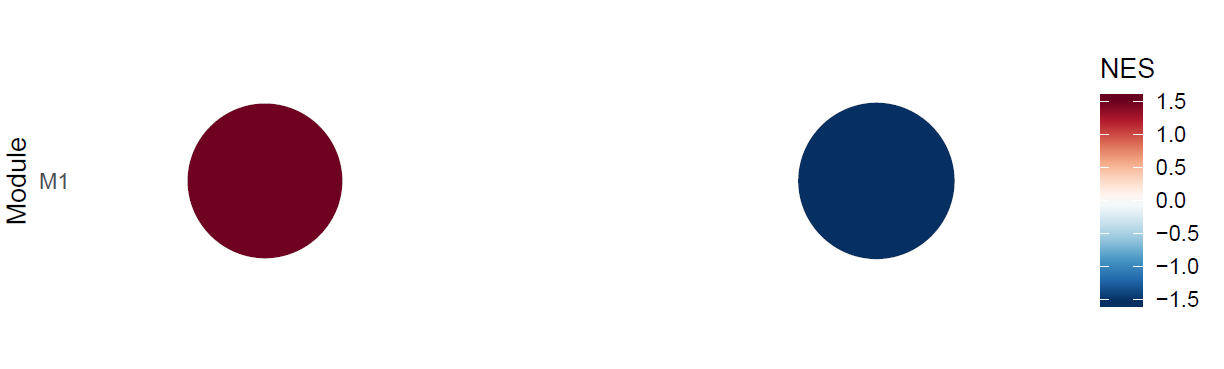

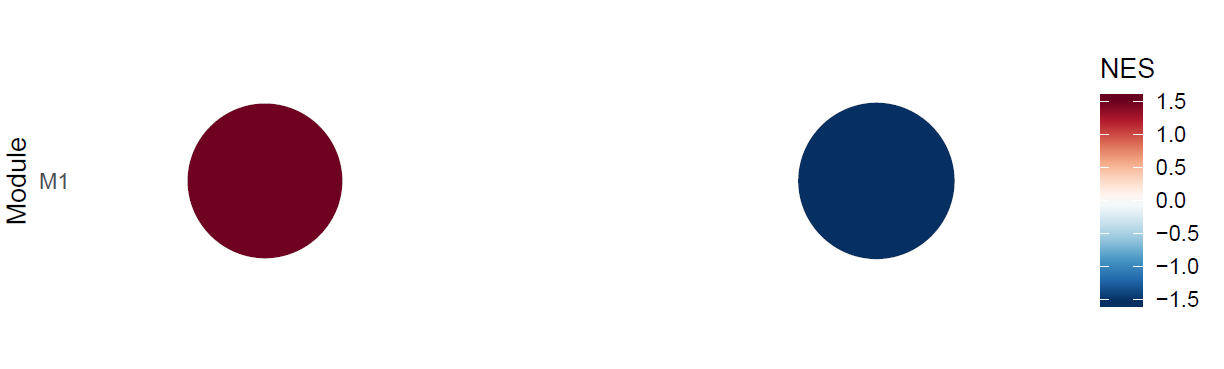

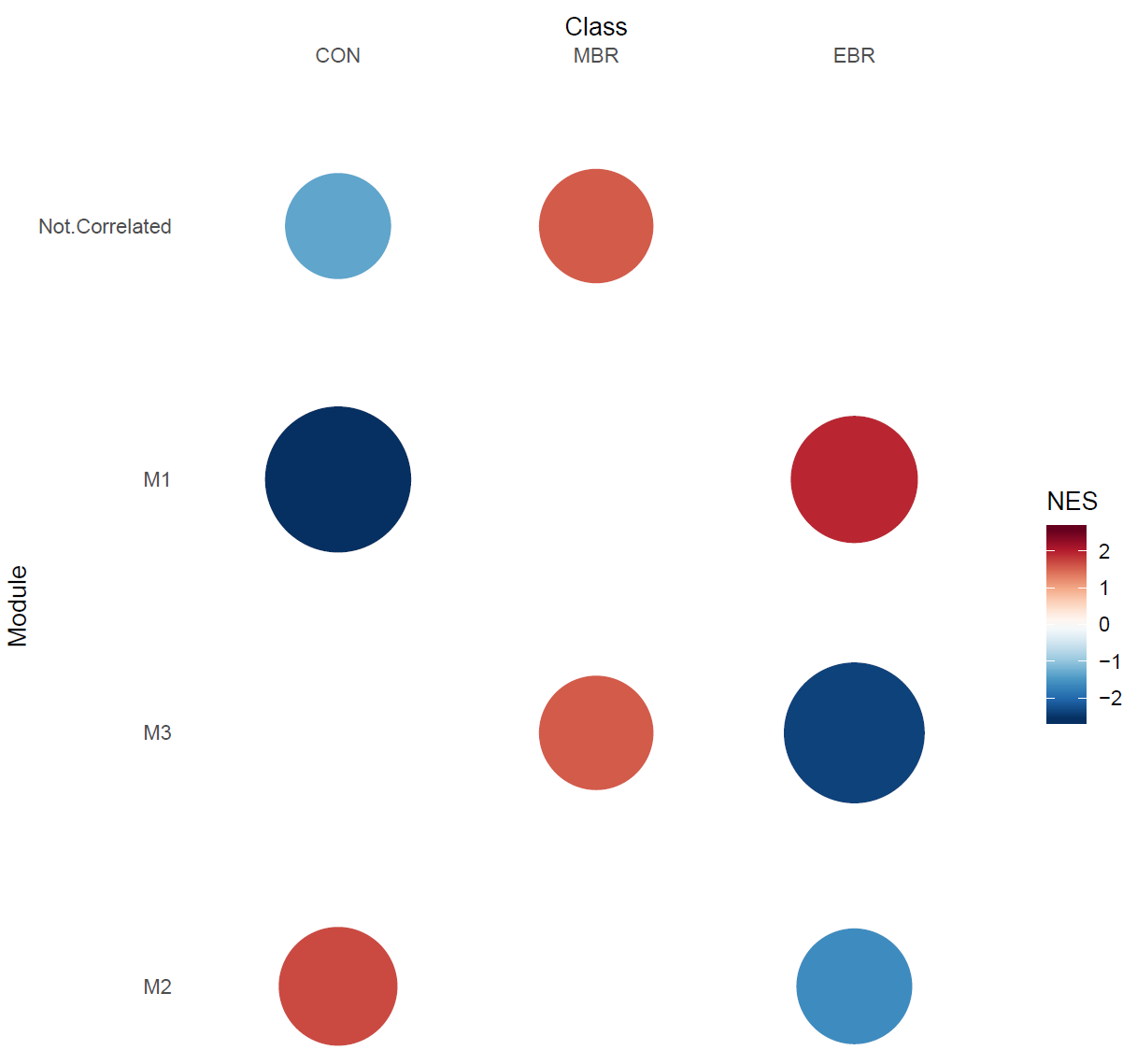

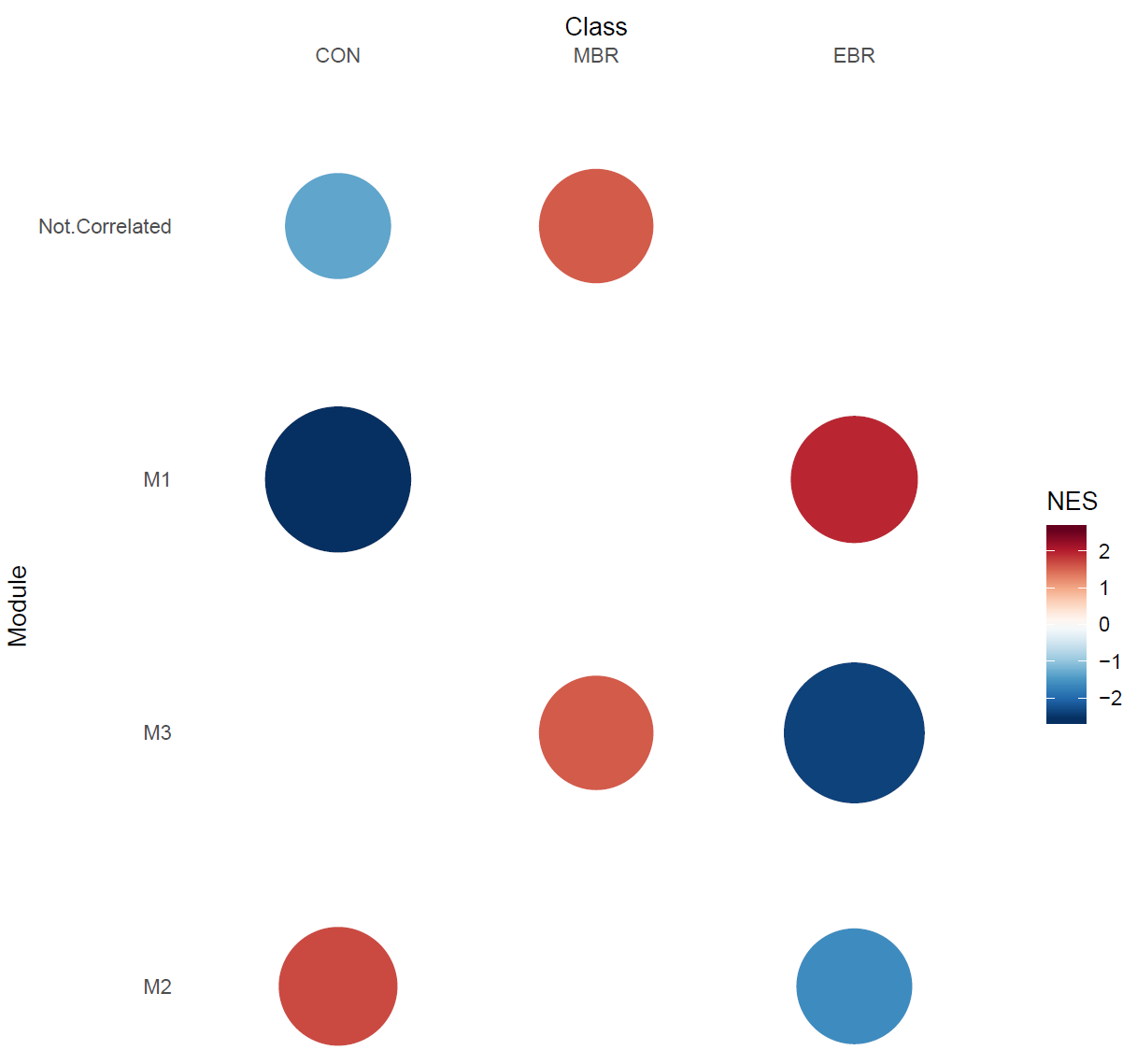


**A**

**B**

Supplement: Supplementary file 1 — Additional file 1. Supplementary methods with details on QC, data processing, and gene annotation steps. [file 13293_2020_288_MOESM1_ESM.zip › Figure S3.docx]
